# Metabolic Adaptation, a Specialized Leaf Organ Structure and Vascular Responses to Diurnal N_2_ Fixation by *Nostoc azollae* Sustain the Astonishing Productivity of *Azolla* Ferns without Nitrogen Fertilizer

**DOI:** 10.3389/fpls.2017.00442

**Published:** 2017-03-31

**Authors:** Paul Brouwer, Andrea Bräutigam, Valerie A. Buijs, Anne O. E. Tazelaar, Adrie van der Werf, Urte Schlüter, Gert-Jan Reichart, Anthony Bolger, Björn Usadel, Andreas P. M. Weber, Henriette Schluepmann

**Affiliations:** ^1^Molecular Plant Physiology, Institute of Environmental Biology, Utrecht UniversityUtrecht, Netherlands; ^2^Institute for Plant Biochemistry, Cluster of Excellence on Plant Sciences, Heinrich Heine UniversityDüsseldorf, Germany; ^3^Plant Research InternationalWageningen, Netherlands; ^4^Organic Geochemistry, Utrecht UniversityUtrecht, Netherlands; ^5^Institute for Botany and Molecular Genetics, Bioeconomy Science Center, RWTH Aachen UniversityAachen, Germany

**Keywords:** dinitrogen fixation, heterocystous cyanobacteria, aquatic ferns, vasculature, diel cycling, clock evolution, *Azolla/Nostoc azollae* symbiosis, RNA-seq

## Abstract

Sustainable agriculture demands reduced input of man-made nitrogen (N) fertilizer, yet N_2_ fixation limits the productivity of crops with heterotrophic diazotrophic bacterial symbionts. We investigated floating ferns from the genus *Azolla* that host phototrophic diazotrophic *Nostoc azollae* in leaf pockets and belong to the fastest growing plants. Experimental production reported here demonstrated N-fertilizer independent production of nitrogen-rich biomass with an annual yield potential per ha of 1200 kg^−1^ N fixed and 35 t dry biomass. ^15^N_2_ fixation peaked at noon, reaching 0.4 mg N g^−1^ dry weight h^−1^. *Azolla* ferns therefore merit consideration as protein crops in spite of the fact that little is known about the fern’s physiology to enable domestication. To gain an understanding of their nitrogen physiology, analyses of fern diel transcript profiles under differing nitrogen fertilizer regimes were combined with microscopic observations. Results established that the ferns adapted to the phototrophic N_2_-fixing symbionts *N. azollae* by (1) adjusting metabolically to nightly absence of N supply using responses ancestral to ferns and seed plants; (2) developing a specialized xylem-rich vasculature surrounding the leaf-pocket organ; (3) responding to N-supply by controlling transcripts of genes mediating nutrient transport, allocation and vasculature development. Unlike other non-seed plants, the *Azolla* fern clock is shown to contain both the morning and evening loops; the evening loop is known to control rhythmic gene expression in the vasculature of seed plants and therefore may have evolved along with the vasculature in the ancestor of ferns and seed plants.

## Introduction

Large yield increases have been achieved by selecting crop varieties particularly responsive to fertilizer applications (Evenson and Gollin, 2003). Yet nitrogen use efficiency of crops when inorganic nitrogen salts are applied to soil is low: as much as 50% of the applied fertilizer is not utilized resulting in often unwanted eutrophication of surrounding ecosystems. Chemical synthesis of nitrogen (N) fertilizer is presently consuming 1–2% of the yearly fossil fuel derived energy and thus is a major contributor to CO_2_ pollution (Erisman et al., 2008). In addition, denitrification of the wasted N-fertilizer leads to release of N_2_O, with greenhouse warming effects up to 300-fold those of CO_2_ (Galloway et al., 2004). Taken together, although spectacular yield increases obtained during the past century with most crops are founded on N-fertilizers, their extensive use is unsustainable, and alternatives must be found to mitigate environmental impact of agriculture.

Reducing N-fertilizer applications without yield penalty may be possible with improved nitrogen use efficiency of crops combined with a more precise application of N-fertilizer (Xu et al., 2012) or by using crops fixing N_2_ from the atmosphere. N_2_ fixation is generally carried out by bacteria associated with the plants, as in root nodules of leguminous crops and in extracellular spaces of sugar cane, or by bacteria freely living in the soil and waterways (Dos Santos et al., 2012; Pankievicz et al., 2015). The most established N_2_-fixing crop symbiosis is the soybean (*Glycine max*) reaching 230 million metric tons annual production in 2008 ranking fourth in production area after wheat, rice, and maize (Hartman et al., 2011). Soybean harbors rhizobia which, after a complex communication between plant and bacteria is triggered, live in nodules of plant roots in facultative symbiosis (Freiberg et al., 1997). Rhizobia in the nodules are fed by plant sugars and secrete ammonium in return (Udvardi and Poole, 2013). Soybean plants reject colonization by rhizobia if nitrogen fertilizer is available. Modern high-yield cultivars, are generally more responsive to N-fertilizer which contributes some 40% to protein in the seed (Wilson et al., 2014). Similar trends are found with the high yielding N_2_-fixing forage alfalfa (*Medicago sativa*), that yields 21 t ha^−1^ a^−1^ dry weight (DW) upon addition of 150 kg N ha^−1^ a^−1^ (Anglade et al., 2015). To further emancipate plant protein production from N-fertilizer, fixation rates of symbionts in established crops will need to be improved (Oldroyd and Dixon, 2014). Alternatively, plant/bacteria symbioses that fix N_2_ more efficiently will need to be domesticated. The latter will counter threats associated with increasingly reduced biodiversity on farm land (Stamp et al., 2012). Development of new or orphan crops is timely due to recent advances in genetics that tremendously speed up breeding.

N_2_-fixing ferns from the genus *Azolla* have been known as bio-fertilizer in submerged field based taro and rice cultivation in China, Vietnam, and Senegal (Shi and Hall, 1988; Wagner, 1997); *Azolla* species distribution is global from temperate to tropical regions. Drained fields emit CO_2_ (Liu et al., 2013). Submerging fields and growing *Azolla* may contribute to reducing CO_2_ emissions. *Azolla* is much easier to contain and harvest in such field situations compared with microalgae or cyanobacteria since growing it does not require sophisticated pumping or mixing to ensure carbonate availability. The N_2_ fixation of these ferns is generally thought to be carried out by the phototrophic bacterium *Nostoc azollae*, which was reported to have an eroded genome as a consequence of its symbiotic lifestyle (Ran et al., 2010). The notion that *N. azollae* are the only symbionts in *Azolla* has been challenged based on electron microscopic observations and this raises questions concerning their role in N trafficking within the symbiosis (Carrapiço, 1991; Zheng et al., 2009). *Azolla/Nostoc* symbioses have probably evolved some 90 M years ago (Metzgar et al., 2007), before the legume/rhizobia symbioses dated to have evolved 64 M years ago (Herendeen et al., 1999; Kistner and Parniske, 2002). *N. azollae* are transmitted vertically during the fern life cycle and thrive inside specialized leaf pockets of the fern that close fully upon maturation after motile hormogonia have infected young leaves (Kaplan and Peters, 1981; Rai et al., 2000; Zheng et al., 2009). Leaves of *A. filiculoides* are arranged in two rows, each leaf has two lobes, the lower lobe is shaped like a boat and rests on the water surface holding the other upper lobe in an aerial position exposed to light (Supplementary Figure 1). The upper leaf lobe contains a leaf pocket with a single ad-axially located pore (Supplementary Figure 1) presumed to mediate exchange of gasses (Veys et al., 1999).

It is hypothesized that the fern contributes sugars while *N. azollae* releases some 40% of the fixed nitrogen into the leaf pocket as ammonia; a reduced glutamate synthase activity in the symbiont compared to free-living cyanobacteria may cause this release (Peters and Meeks, 1989; Meeks, 2009). The ammonia is then assimilated by the glutamate synthase and glutamine synthetase (GS/GOGAT) cycle of the fern (Meeks et al., 1987). Label from ^15^N_2_ fixed travels from the mature leaves to the shoot tips presumably supplying young leaves and dividing bacterial cells of the shoot tip (Ito and Watanabe, 1983). Molecular mechanisms underpinning the fern responses to N supplied by *N. azollae* are entirely unknown, yet a gene database was recently established from transcriptome sequencing that may now be used to profile the responses (Brouwer et al., 2014). *Azolla* species are known to thrive in ditches containing nitrate or/and ammonium, possibly in environments with high P/N ratios. ^15^N-labeled nitrate is known to be taken up by the root and the label to move slowly to the shoot where it accumulates in the old branches. In contrast, ^15^N-labeled ammonium is more rapidly taken up and its label rapidly accumulates in the shoot tip, more rapidly that from ^15^N_2_ (Ito and Watanabe, 1983). The difference suggests that nitrate reduction occurs at a low rate in the fern. In *Azolla* unlike in many cyanobacteria, low concentrations (1 mM) of NH_4_SO_4_ or potassium nitrate did not inhibit nitrogenase assayed using the acetylene reduction assay which suggests that cyanobacteria in *Azolla* may not be exposed to medium concentrations of nitrogen salts (Ito and Watanabe, 1983). Particularly nitrate salts did not inhibit nitrogenase activity until 25 mM in 24 h exposure. This could be due to absence of nitrate transporters in *N. azollae* as reported for *A. filiculoides* (Ran et al., 2010).

In batch cultures illuminated with incandescent light, *Azolla* ferns from several species were reported to reach doubling times as low as 2 days in their exponential growth phase without N-fertilizer, yielding an N-content of 4–5% of the DW (Peters et al., 1980). Due to high productivity the ferns ought to be considered as high protein crops in their own right. Data on productivities without N-fertilizer, however, is lacking for ferns grown in the linear growth phase in closed-canopy cultures, the latter being crucial to suppress algae and outgrow fungal and insect pests.

Here we set out to examine the productivity of *A. filiculoides* under continuous production conditions over 138 days. The effect of various inorganic N-fertilizers on the productivity in terms of biomass, the N-content of the biomass and expression of key fern and bacterial marker genes were analyzed simultaneously. Confocal laser scanning microscopy was used to reveal features of the leaf pocket important for metabolic connectivity of the symbiosis. *A. filiculoides* clones with and without *N. azollae* were used to verify the contribution of *N. azollae* to the fern growth rates. ^15^N_2_-fixation rates were measured during the diel cycle and after acclimation to 2 mM NH_4_NO_3_. Diel transcriptome analysis was used to probe the fern diel physiological response to different nitrogen sources. RNA sequence assemblies were further used to examine ammonium transporters and markers of vasculature development.

## Materials and Methods

### Plant Materials

*Azolla filiculoides* Lam. was collected in Utrecht as described by Brouwer et al. (2014). *A. pinnata* R. Br. was from the International Rice Research Institute Bio-fertilizer Collection [code PI 0535, originally from Sigiriya in Sri Lanka provided by S. Kulasooriya in 1984; (Watanabe et al., 1992)].

### Growth Conditions

The Standard *Azolla* Medium (SAM) was liquid IRRI medium (Watanabe et al., 1992) with the following modifications: 0.32 μM, CuSO_4_, 0.835 μM ZnSO_4_, and 17.9 μM Fe-EDTA. The medium was neither pumped, nor mixed during growth. Light intensity was 300 μmol photons m^−2^ s^−1^ over the wave band 400–700 nm supplemented with incandescent lamps for 12 h. Day-time temperature was 22°C whilst night-time temperature was 15°C with the exception in the ^15^N_2_-fixation experiment where temperature was kept a constant 21°C. The growth conditions under experimental production were as above but incandescent lamps were turned on for only 2 h at the end of the day. Containers were open to ambient air; water was stagnant. Mat density reached 2–3 kg m^−2^ fresh weight (FW) biomass before the continuous harvest under linear growth commenced; after each harvest nutrients were added to replace the nutrients removed in the biomass harvested. Full medium replacement was carried out once every 5 weeks.

To obtain sterile cultures of the symbiosis, frond pieces (<1 mm^3^) of *A. filiculoides* were surface sterilized using bleach at 1% available chlorine for 40 s and four consecutive rinses in sterile water before culture on agar solidified SAM medium. SAM was solidified with 0.6% agarose (Duchefa, Netherlands). Fern clones without *N. azollae* were selected on agar solidified SAM with 60 μg ml^−1^ Erythromycin as previously described (Forni et al., 1991); absence of *N. azollae* was routinely verified by confocal microscopy using the characteristic bacterial fluorescence as a marker (Brouwer et al., 2014).

Sterile cultures of surface sterilized plants (1 L liquid medium with and without 2 mM NH_4_NO_3_) were grown in enclosed glass containers with a stream of air (78 L h^−1^) pumped through 0.45 μm filters using aquarium pumps (SuperFish Air flow mini); these sterile cultures were used in all of the DNA and RNA sequencing experiments. Sterile cultures were also used for the ^15^N_2_-fixation experiments.

### Confocal Laser Scanning Microscopy

Propidium iodide/periodic acid staining and mounting of the tissues were essentially as described by Truernit et al. (2008). A Leica SP2 laser scanning confocal fluorescence microscope, equipped with either ×10 or × 40 objectives and a laser with excitation wavelength of 405 nm, was used to visualize propidium iodide at 530–560 nm and auto-fluorescence at 515–530 nm.

### RNA Extractions and Quantitative RT-PCR

Plant material was dry blotted, 50 mg FW snap frozen in liquid N_2_, then ground frozen with two glass beads using the TissueLyser II (Qiagen) set at maximum speed for 1.5 min twice. RNA was extracted with the Spectrum Plant Total RNA kit applying protocol B (Sigma–Aldrich). RNA was then treated with DNase (5 units for 3 μg RNA) 30 min at 37°C and the reaction stopped by 10 min at 65°C in the presence of EDTA (2 mM).

Primers (polyT and random hexamers at 0.030 and 0.074 μg μl^−1^ final concentrations, respectively, in the reverse transcription reaction) and 1 μg DNase treated RNA were denatured 5 min at 72°C before reverse transcription with MLV reverse transcriptase (5 units, Fermentas) for 10 min at 37°C then 40 min at 42°C. Primers used for q-RT PCR are listed in Supplementary Table 1. Quantitative RT-PCR was performed as described in Brouwer et al. (2014).

### RNA Sequencing and Bioinformatic Analyses

Surface sterilized *A. filiculoides* were grown in excess sterile SAM with and without 2 mM NH_4_NO_3_ for 7 days in triplicate replicate cultures. Growth was in a chamber with light set to start at 0600 for 12 h. On day 8 samples were collected, snap frozen at 2, 8, 14, and 20 h of the diel cycle, then total RNA extracted and DNAse treated as above. RNA integrity, sequencing library quality, and fragment size were verified on a 2100 Bioanalyzer (Agilent). Libraries were prepared using the TruSeq RNA Sample Prep Kit v2 (Illumina), and library quantification was performed with a Qubit 2.0 (Invitrogen). All libraries were sequenced on the HISEQ2000 Illumina platform in paired-end mode.

Paired-end reads including public available reads from de Vries et al. (2016) were mapped against the transcriptome database (Brouwer et al., 2014) using CLC genomics workbench^®^(CLC bio, Denmark) using default parameters (mismatch cost 2, insertion cost 3, deletion cost 3, length fraction 0.8, similarity fraction 0.8, auto-detect paired distance, strand specific both and maximum number of hits for a read 30). Only paired matches were counted. Read counts were used for statistical analysis with edgeR in classic mode (Robinson et al., 2010) using the Bioconductor package. Results were corrected for multiple hypothesis testing using Benjamini–Hochberg FDR correction (Benjamini and Hochberg, 1995) and considered significant if *q* < 0.01. Read counts were normalized to total mapped reads for each sample and expressed as reads per million reads (rpm; Supplementary Tables 2, 4). Principle component analysis and clustering were conducted on all transcripts which being summed exceeded 10 rpm using the MultiExperimentViewer^1^.

Clock genes were annotated as the closest homolog to the *Arabidopsis thaliana* clock genes (Nakamichi, 2011). The diel transcripts were identified by isolating transcripts which display differential expression between at least one pair of two adjacent time points then loaded into Mapman for visualization and enrichment tests (Thimm et al., 2004). Functional annotations were imported using the Mercator Pipeline (Lohse et al., 2014) based on the *Azolla* transcriptome sequences (Brouwer et al., 2014). K-means clusters were built using the MultiExperimentViewer with Pearson distance metric.

### N-Content Determinations and ^15^N_2_ Fixation Rates

Ferns (100 mg FW) were grown in pots with 43 ± 4 ml of sterile SAM and a remaining air space of 262 ± 4 ml. To the air space 25 ml of ^15^N_2_ was added using air-tight syringes; overpressure was slowly brought to ambient pressure using a release needle. Plants were subsequently grown in these conditions for 2 h at the following time points: 8, 14, 20, 2, and 8 h in the diel cycle where the 12 h day started at 7 h. Ferns devoid of cyanobacteria at the 14 h time point, in the light, were used as the no fixation control. In addition, the effect of 7 day acclimation to 2 mM NH_4_NO_3_ on ferns with cyanobacteria was tested at the 14 h time point. Samples were frozen at −20°C, homogenized and freeze-dried before analysis of the dry weights and isotope abundance determinations. All data points represent the average of triplicate biological samples.

Total N-content and stable nitrogen isotopes (δ^15^N) were analyzed on a ThermoScience Delta Plus isotope ratio mass spectrometer connected on-line to a Carlo Erba Instruments Flash 1112 elemental analyzer. The δ^15^N of each sample was expressed relative to atmospheric dinitrogen as (‰) and calculated as the following ratio: (([^15^N_Sample_]-[^15^N_Air_]) ^∗^ 10^3^)/[^15^N_Air_]) with [^15^N_Sample_] the concentration of ^15^N isotope in the sample, [^15^N_Air_] the concentration of ^15^N in standard air. Instrumental precision was better than 0.2‰. N_2_ fixation rates were determined using the following calculation: ((([^15^N_t2_] [N_biomast2_])-([^15^N_t0_] [N_biomast0_]))/[^15^N_Airspace_] ^∗^ 2 h) with [^15^N_ti_] the concentration of ^15^N before (*i* = 0) or after 2 h incubation (*i* = 2) with ^15^N_2_ enriched air in the bottle, [N_biomasti_] the N-content in the biomass before (*i* = 0) and after 2 h incubation (*i* = 2) and [^15^N_airspace_] the ratio of ^15^N over total N in the air space. We assumed no isotope discrimination during the fixation process and therefore rates of fixation calculated may be underestimated.

## Results

### *Azolla filiculoides* Yielded 35 t DW ha^−1^ a^−1^ in an Experimental Continuous Harvest System

**FIGURE 1:**
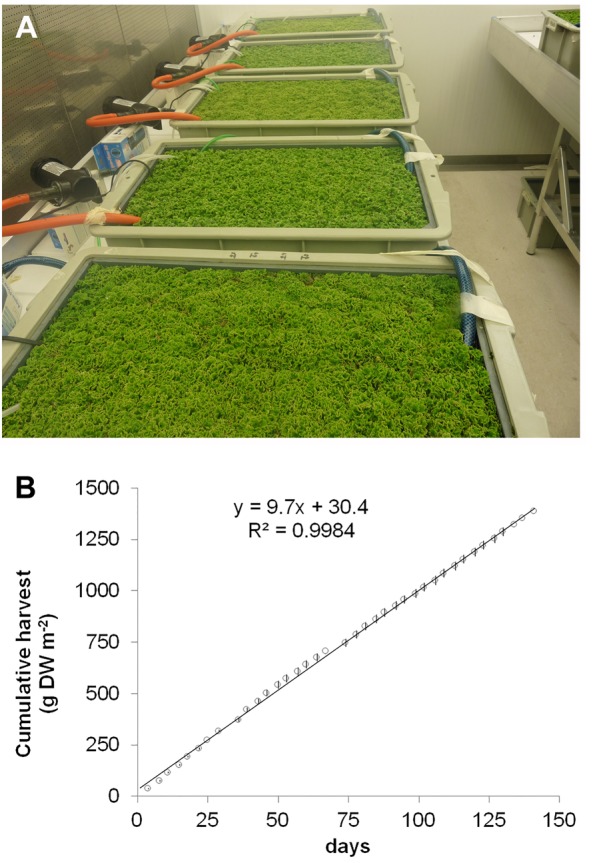
***Azolla filiculoides* yield potential under continuous production conditions.** Ferns were grown with 300 μmol s^−1^ cm^−2^ photosynthetic active radiation at 25°C for the 12 h day and at 22°C during the 12 h night. **(A)** Closed-canopy cultures with plants in the linear growth phase for continuous harvest. **(B)** Cumulative dry biomass harvested. Once standing crop formed a closed mat at 160 g m^2^ dry weight, continuous harvest was at a rate of 33% of the biomass twice per week. Linear regression equation is shown along with its fit; *n* = 6, standard deviations were smaller than the labels.

**FIGURE 2:**
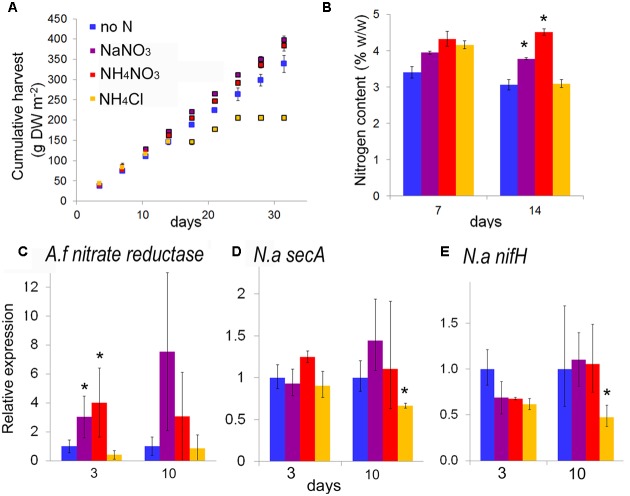
***Azolla filiculoides* response to N-fertilizer under continuous production conditions.** Ferns were grown as in **Figure 1** but this time in medium without (no N) or with 4 mM NaNO_3_, or 2 mM NH_4_NO_3_, or 4 mM NH_4_Cl. Biomass harvested was split to determine cumulative harvest **(A)** nitrogen content **(B)** and to extract mRNA. Reverse transcription of the RNA was with an excess random hexamers to analyze levels of *A. filiculoides* and *Nostoc azollae* transcripts by quantitative PCR: *A. filiculoides* nitrate reductase Afcontig 35782 **(C)**, *N. azollae secA* **(D)** and *nifH* **(E)**. Standard deviation is for *n* = 3, *T*-test significance with reference to no N for *P* < 0.05 is marked with ^∗^.

To evaluate the potential of species from the genus *Azolla* as high protein biomass crops, *A. filiculoides* was grown under continuous production conditions for 138 days (**Figure 1A**). When harvesting 33% of the surface twice per week, accumulated biomass increased linearly (**Figure 1B**). From the slope of dry biomass accumulation, productivity was calculated at 9.72 g biomass DW m^−2^ day^−1^, corresponding to an annual yield potential of 35.5 t ha^−1^ DW for *A. filiculoides*. Very similar results were obtained with *A. pinnata* (data not shown). To test whether yield performance of these ferns was influenced by N-fertilizers often encountered in runoff from agricultural land, we examined the effect of several inorganic nitrogen sources in the media used for continuous production. Growth of *A. filiculoides* was in SAM without nitrogen or with 4 mM NaNO_3_, NH_4_Cl or 2 mM NH_4_NO_3_. We harvested 33% of the culture area twice weekly over a period of 4 weeks, whilst replenishing nutrients weekly. Cumulative harvest over 30 days indicated slight benefits with N-fertilizer (up to 15%, **Figure 2A**). N-fertilizers moreover led to N accumulation in the biomass, from 3.41 up to maximally 4.36% w/w N with NaNO_3_ (**Figure 2B**) and to induced expression of nitrate reductase from the fern (**Figure 2C**). NaNO_3_ as well as NH_4_NO_3_ did not affect transcript abundance of the bacterial housekeeping gene *N. azollae secA* suggesting that bacterial growth was not affected by NO_3_^−^ containing media (**Figure 2D**). In contrast, NH_4_Cl reduced *A. filiculoides* productivity and led to a reduction of *N. azollae secA* and *nifH* transcript detection (**Figures 2D,E**). *nifH* encodes the catalytic subunit of the nitrogenase enzyme and is intact in *N. azollae* (Ran et al., 2010). The above results demonstrate that *A. filiculoides* does not require N-fertilizer to sustain high productivity but that it reacts to and benefits from additional N sources in the medium.

### Growth of *A. filiculoides* without *N. azollae* Entirely Relied on Exogenous N-Fertilizer

**FIGURE 3:**
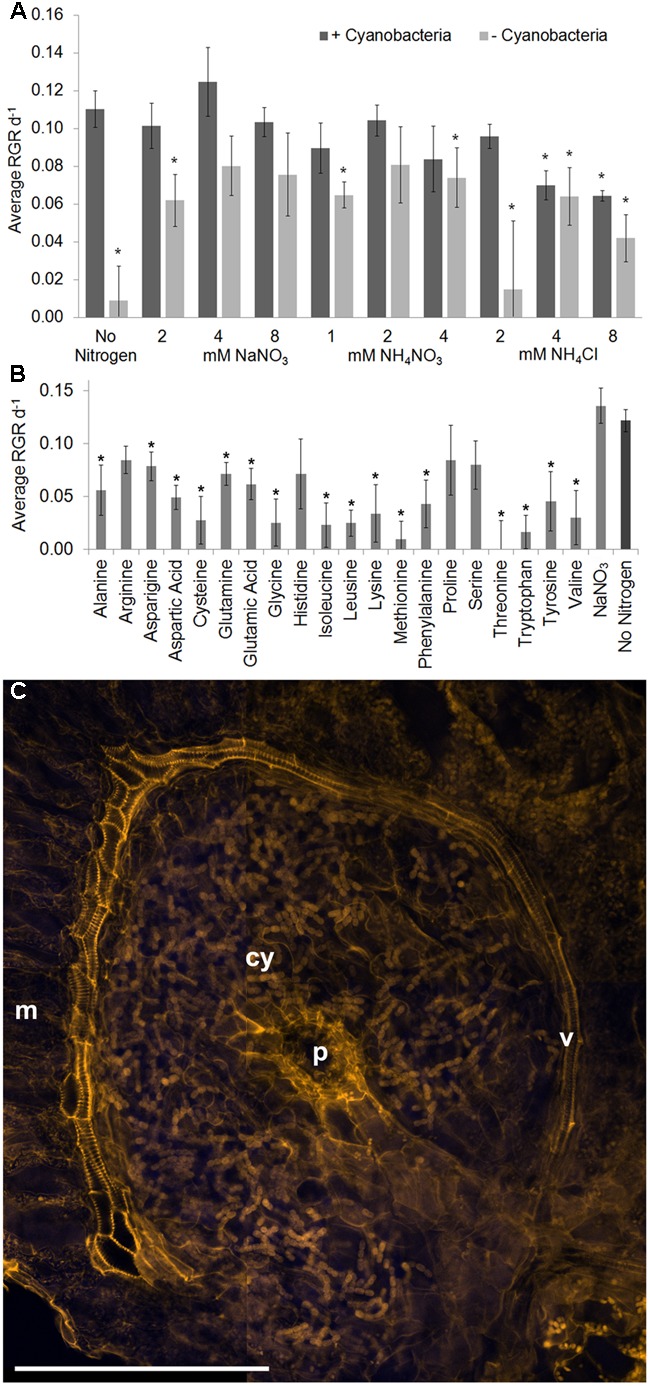
**Role of *N. azollae* in sustaining fern growth and specific structures of the leaf pocket.** Clonal ferns with (+cyano) and without *N. azollae* (-cyano) were grown on agar solidified Standard *Azolla* Medium (SAM). **(A)** Growth in inorganic N-fertilizer ranging from 2 to 8 mM. **(B)** Growth with 2 mM nitrogen in differing amino acids, or with 4 mM NaNO_3_ (NO_3_), and +cyano on SAM for comparison. Growth was measured as leaf area. Ferns without *N. azollae* failed to grow on SAM but in all other conditions growth fitted an exponential curve. Standard deviations with *n* = 3, *T*-test significance with reference to ferns +cyano on SAM for *P* < 0.05 is marked with ^∗^. **(C)** Structures revealed by propidium iodide staining using confocal microscopy 405 nm excitation, 560 nm emission (orange); auto-fluorescence at 515–530 nm emission (blue). v, vasculature; p, pore; cy, *N. azollae* bacteria; m, mesophyll cells; scale bar: 200 μm.

To evaluate the contribution from *N. azollae* to fern productivity we raised surface sterilized *A. filiculoides*, then for every fern, fronds were grown without and with erythromycin so as to obtain clonal material with and without cyanobacteria. Complete absence of *N. azollae* was verified using confocal microscopy and quantitative PCR on the extracted plant DNA (Supplementary Figure 2). *A. filiculoides* without *N. azollae* failed to grow without a source of reduced N and with 2 mM NH_4_Cl (**Figure 3A**). Growth in all other conditions was exponential. Optimum supplementation of ferns without *N. azollae* was using 4 or 8 mM NaNO_3_, or 2 mM NH_4_NO_3_, and yielded growth rates approaching those of ferns with *N. azollae* (**Figures 2A**, **3A**). Given the possibility of amino acids as exchange substrates between fern and symbiont in addition to ammonia (Kaplan and Peters, 1981; Meeks et al., 1987), amino acids were tested for their ability to restore growth keeping the total N supplied in the amino acids at 2 mM in the medium (**Figure 3B**). Whilst arginine and proline supported highest average growth rates that were not significantly different from ferns with *N. azollae*, growth rates on amino acids mostly correlated with those from the seed plant *A. thaliana* (Forsum et al., 2008) and, therefore, unlikely reflected trafficking inside leaf pockets. Close-up inspection confirmed that the leaf pockets were well removed from the medium and enclosed entities except for the leaf-pocket pore open to air (**Figure 3C** and Supplementary Figure 1). Confocal microscopy further revealed a conspicuous vasculature curving around the pockets that was closely connected to the pocket fluid (**Figure 3C**). This vasculature differed from that in leaf lobes without leaf pocket: it was made of several layers of tracheid cells with re-enforced cell walls containing pectin brightly stained by the propidium iodide/periodic acid stain (Supplementary Figure 3).

### N_2_-Fixation Rates Reached 0.4 mg N g^−1^dw h^−1^ at Noon and Dropped 7.5 Times in the Dark

**FIGURE 4:**
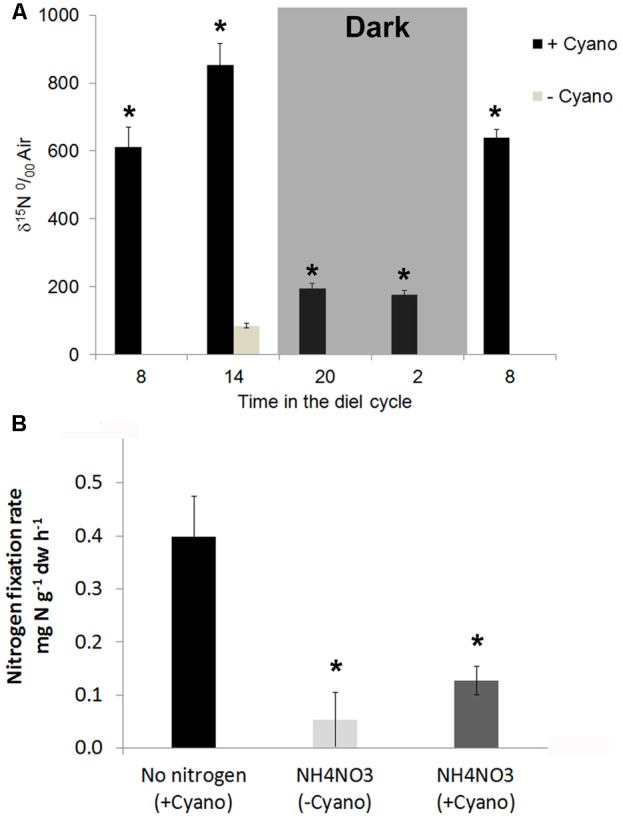
**^15^N_2_ fixation during the diel cycle and after acclimation to 2 mM NH_4_NO_3_. (A)** Raw ^15^N enrichment after 2 h exposure to ^15^N_2_ averaged for the time points 8, 14, 20, and 2h during the diel cycle. Ferns were on SAM without nitrogen, the 12 h day started at 6 h. Temperature was kept a constant 21°C. Ferns with *N. azollae* (+Cyano, black) and without (–Cyano, light gray). **(B)** ^15^N_2_-fixation rates averaged over 2 h at the timepoint 14 h in the diel cycle after a week acclimation on SAM without [No nitrogen (+Cyano), black and with 2 mM NH_4_NO_3_ (NH_4_NO_3_ (+Cyano), dark gray]; control ferns without *N. azollae* [NH_4_NO_3_ (–Cyano), light gray]. Standard deviations with *n* = 3. *T*-test significance marked with ^∗^ was *P* < 0.05 for +Cyano data points with reference to ferns –Cyano on SAM for **(A)**; and with reference to +Cyano ferns on no N in **(B)**.

*Nostoc azollae* are phototrophic bacteria, therefore, we set out to measure diel ^15^N_2_ fixation in *Azolla*. We first established that N_2_-fixation rates were constant over at least 12 h when keeping light, temperature, and CO_2_ concentrations constant (data not shown). Ferns in excess medium were exposed to ^15^N_2_-enriched air for 2 h starting at 8, 14, 18, 20, and 8 h in the diel cycle of a growth chamber with 12 h light starting at 7 h. Ferns without *N. azollae* constituted the control non-fixing ferns at the 14 h time point. The relative enrichment of ^15^N over air (δ^15^N) in *Azolla* biomass at 20 and 2 h in the dark was up to 7.5 times lower than that measured at noon (**Figure 4A**). *N. azollae*, therefore, fixed the bulk amounts of N_2_ during day-time.

We further assessed ^15^N_2_-fixation rates in ferns acclimated for a week to +N (2 mM NH_4_NO_3_) or –N (SAM) during peak N_2_ fixation at 14 h (**Figure 4B**). Whilst the N_2_-fixation rate in ferns grown without nitrogen reached 0.394 mg N g^−1^ DW h^−1^, N-fertilizer reduced ^15^N_2_-fixation rates fourfold. The dramatic change further indicated that responses studied in ferns +N resulted from N-uptake rather than the combination of fixation and uptake.

### Transcriptional Patterns of the Diel Cycle Dominate over the Response to N-Fertilizer

**FIGURE 5:**
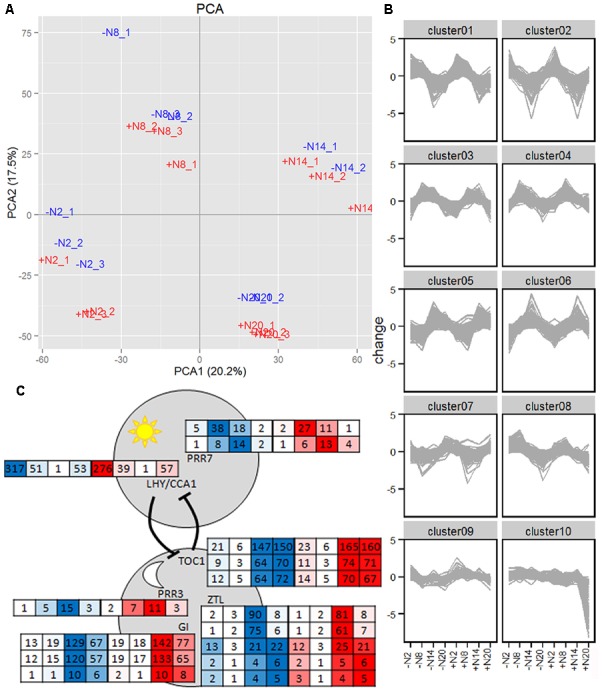
**Diel transcript profiles in *A. filiculoides* grown with and without 2 mM NH_4_NO_3_.** Ferns were acclimated to SAM without (–N, in blue) and with 2 mM NH_4_NO_3_ (+N, in red) for a week then harvested from triplicate cultures at 2, 8, 14, and 20 h with 12 h day-light starting at 6 h. RNA was extracted, sequenced and sequences analyzed as described in the section “Materials and Methods.” **(A)**, Principal Component Analysis (PCA) and **(B)**, K-means clustering included all genes with a read count > 10 rpm for all time points and conditions. K-means clusters were built with Pearson distance metric. **(C)** Clock components from the morning (above) and evening (below) loops with their respective read counts in rpm, on medium without N (in blue) and with N (in red) at 2, 8, 14, and 20 h in each box, respectively.

The fern’s ability to react to N-fertilizer prompted an analysis of the transcriptional mechanisms underpinning these responses; the specificity with which cDNA libraries were generated afforded stringent spatial resolution thus distinguishing for the first time processes in the fern from those in the cyanobacteria. Given the diel behavior of nitrogen assimilation genes in the angiosperm *A. thaliana* (Cheng et al., 1991; Gutiérrez et al., 2008), we chose to compare the diel transcript profiles of ferns acclimated for a week to SAM without nitrogen (-N) or with 2 mM NH_4_NO_3_ (+N). Ferns were sampled from triplicate cultures at 2, 8, 14, and 20 h within the 24 h cycle of a growth cabinet with 12 h light starting at 6 h in the morning. RNA-seq resulted in 17.3–37.8 million read pairs per sample of which between 47–55% mapped as read pairs and 63–65% mapped as single end reads to the transcriptome database [Brouwer et al., 2014; Supplementary Table 2_mapping_stats]. Transcriptional investments in functional categories were similar in *A. filiculoides* and *A. thaliana* (Supplementary Figure 4). Principle component analysis indicated four groups that corresponded to the different time points during the day which separated in both the first and second dimension and explained 38% of the variation in the dataset (**Figure 5A**). The first two dimensions also resolved the N-fertilized samples: +N samples projected apart from the –N samples in the same direction at all time points.

Diel transcripts were defined as transcripts with read counts significantly changed in at least two adjacent time points in either the +N or –N series. Of all transcripts detected, 4872 or 13% were diel (Supplementary Table 2_diel_transcripts). K-means clustering of diel genes identified clusters (**Figure 5B** and Supplementary Table 2_clusters) with genes peaking at 2 h (8 h into the dark, clusters 1 and 2), genes peaking at 8 h (2 h into the light, clusters 3 and 4), genes peaking at 14 h (8 h into the light, clusters 5 and 6), and genes peaking at 20 h (2 h into the dark, cluster 7) with no difference between the –N and +N samples, indicating that diel rhythms of transcription were unresponsive to N-fertilizer, with the exception of genes in cluster 10 (369 contigs) that sharply decreased 2 h into dark if N was supplemented (**Figure 5B** and Supplementary Table 2_cluster_10). Cluster 10 genes included genes from the Calvin-Benson cycle, PS1 and PS2 light harvesting complexes, and glycine decarboxylase P-protein 2 (Af_7035) from photorespiration; in addition it included the amino acid sensor ACT domain repeat 3-like protein (Af_11070) and three sugar transporters (Af_3233, Af_17210, and Af_14354).

Clock-gene expression patterns corresponded to those in *A. thaliana* with the morning loop peaking at 2 and 8 h and the evening loop peaking at 14 and 20 h although the number of isoforms did not correspond to the numbers in *A. thaliana*; clock-gene transcripts did not respond to N-fertilizer (**Figure 5C** and Supplementary Table 2_clock_genes). Diel transcripts were enriched in starch and sucrose metabolism, pathways of photosynthesis, nutrient assimilation and selected pathways in secondary, lipid, and amino acid metabolism (Supplementary Table 3). Diel rhythms of gene expression and clock components in *A. filiculoides* ferns were hence similar to those in angiosperms. In the fern, moreover, diel rhythms of gene expression dominated over the response to N-fertilizer.

### Transcriptional Profiles of Ferns Grown without N-Fertilizer Reflected Early Morning Recovery from Night-Time N Deficiency

**FIGURE 6:**
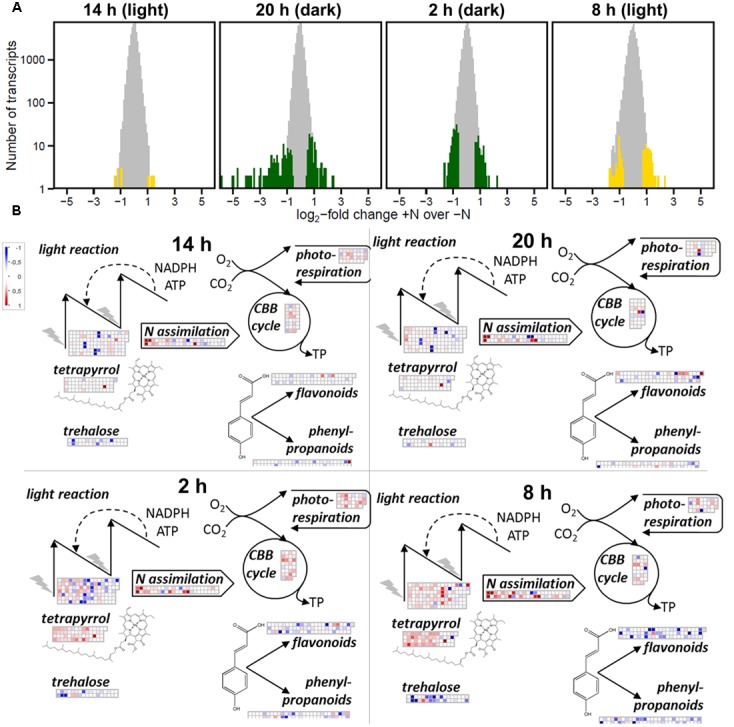
**Metabolic responses of *A. filiculoides* to diurnal supply of reduced N from *N. azollae*.** **(A)** Number of genes differentially transcribed comparing log_2_-fold change of the read-count ratios in ferns with 2 mM NH_4_NO_3_ (+N) over without (–N). Yellow, time points in the light; green, time points in the night. **(B)** Mapman overview of transcriptional changes in metabolism comparing transcription +N over –N. Noon (14 h), evening (20 h; 2 h into dark), night (2 h), and morning (8 h; 2 h into the 12 h day). Upregulation in –N is depicted in red, downregulation is depicted in blue, maximal coloration is set at twofold changes.

Acclimation to +N (2 mM NH_4_NO_3_) compared to –N changed the amounts of 526 transcripts significantly when all contigs were counted that were changed in at least one time point (Supplementary Table 2_N_responsive). The changes were, however, not uniform throughout the day: most changes occurred during darkness at 20 and 2 h with 250 and 240 changed contigs, respectively (**Figure 6A**). In contrast only 28 contigs were changed at noon (14 h; **Figure 6A**).

To visualize N-dependent transcriptional investments in central metabolic pathways, transcript read counts were submitted to the Mapman software [**Figure 6B**; (Thimm et al., 2004)]. In the middle of the night (2 h) and in the morning (8 h), +N conditions led to accumulation of transcripts involved in tetrapyrrole synthesis (*q* < 10^−9^), the Calvin-Benson cycle (*q* < 10^−20^), and in the morning transcripts of the light reaction (*q* < 10^−9^). In contrast, transcripts from flavonoid (*q* < 10^−3^), phenylpropanoid synthesis (*q* < 10^−3^), and trehalose metabolism (*q* < 10^−2^) accumulated in –N conditions, most strongly in the morning at 8 h (**Figure 6B** and Supplementary Table 4). This pattern essentially reflected the pattern reported in N-starved *A. thaliana* upon nitrate addition (Scheible et al., 2004) and therefore suggested N deficit at night in ferns grown in the absence of N.

### Pathways for the Assimilation of N into Amino Acids Remained Unchanged but Ureic Acid Metabolism Changed with N Supply

Transcriptional investment in N-assimilation pathways into amino acids, remained unchanged when comparing +N with –N with very few exceptions: transcripts accumulated for two contigs of glutamine dehydrogenase (Af2747 and Af3257) in ferns on +N but were much less abundant than transcripts of GOGAT (Af4692) and GS (Af31780) enzymes (**Table 1** and Supplementary Figure 5), and therefore may represent enzymes in specific tissues exposed to +N from the medium.

**Table 1:** Transcripts associated with N metabolism that responded to +/-N conditions at the time points 2, 8, 14, or 20 h of the diel cycle.

	Average read abundance in rpm at each time point of the diel cycle								Fold change +/- N^∗^				*Q*-value +N versus –N^∗^			
			
	+N				-N											
Protein	2	8	14	20	N2	N8	N14	N20	2	8	14	20	2vsN2	8vsN8	14vsN14	20vsN20
Allantoate amidohydrolase	475	712	101	57	166	170	73	48	−**1.51**	−**2.06**	−0.46	−0.25	**2.0E-04**	**3.4E-03**	1	1
Allantoate amidohydrolase	85	123	20	11	35	33	13	7	−**1.27**	−**1.88**	−0.56	−0.56	**9.2E-03**	**5.2E-03**	1	3.7E-01
Ammonium transporter 2	4	3	4	4	0	1	0	1	−**1.65**	−**1.25**	−**1.93**	−**1.25**	**1.5E-06**	**1.2E-03**	**2.8E-07**	**3.0E-03**
Urea transporter	69	56	96	69	20	25	50	34	−**1.73**	−1.10	−0.92	−**1.00**	**3.6E-16**	4.1E-02	7.0E-01	**3.9E-03**
Aspartate aminotransferase 5	16	17	19	17	15	10	16	0	−0.11	−0.75	−0.24	−**4.03**	1.0E+00	7.6E-01	1	**4.9E-29**
Ureide permease 2	36	43	30	24	22	26	27	21	−**0.66**	−0.72	−0.16	−0.13	**3.9E-03**	1.1E-01	1	1
Glutamine synthase clone R1	97	128	110	107	113	88	106	1	0.21	−0.54	−0.05	−**5.87**	1	9.9E-01	1	**8.1E-92**
NADH-GOGAT	147	136	153	172	167	153	163	239	0.19	0.16	0.09	**0.47**	8.6E-01	8.1E-01	1	**7.2E-03**
Aspartate aminotransferase 1	18	19	16	21	30	28	17	21	**0.72**	0.55	0.10	0.04	**2.0E-03**	1.1E-01	1	1
Glutamate dehydrogenase 1	20	21	9	16	67	46	25	47	1.69	1.12	**1.42**	**1.50**	**3.0E-11**	1.1E-02	**8.3E-07**	**2.2E-08**
Glutamate dehydrogenase 1	3	4	2	2	14	10	4	10	**1.96**	1.23	0.98	1.78	**2.0E-09**	2.4E-02	1.6E-01	**7.9E-15**
Nitrate reductase 1	24	6	3	8	38	35	22	48	0.64	**2.36**	**2.63**	**2.49**	7.7E-02	**2.8E-11**	**2.2E-14**	**6.3E-45**
Nitrite reductase 1	20	16	18	24	85	141	90	238	**2.01**	**3.03**	**2.22**	**3.27**	**1.5E-14**	**1.5E-15**	**6.6E-11**	**1.4E-33**
Nitrite reductase 1	27	22	24	31	133	199	126	289	**2.25**	**3.11**	**2.36**	**3.16**	**3.5E-14**	**3.5E-23**	**3.1E-17**	**2.8E-38**

^∗^*q-value was corrected for multiple hypothesis testing by Benjamini–Hochberg, significant changes are bold.*

At night in plants grown –N, stored ureides may have been remobilized by allantoate amidohydrolase (Af8889) as reads from this enzyme were abundant. In contrast at night in ferns grown +N, reads of this enzyme were low (**Table 1**). Consistently in ferns grown +N at night, N-remobilization likely was decreased since transcripts of a transporter of urea (Af13203) and ureide permease (Af5356) were fewer whilst urea biosynthesis likely was maintained since transcripts of the aspartate aminotransferase 1 (Af3010) and NADH-dependent GOGAT (Af4692) accumulated (**Table 1**).

**FIGURE 7:**
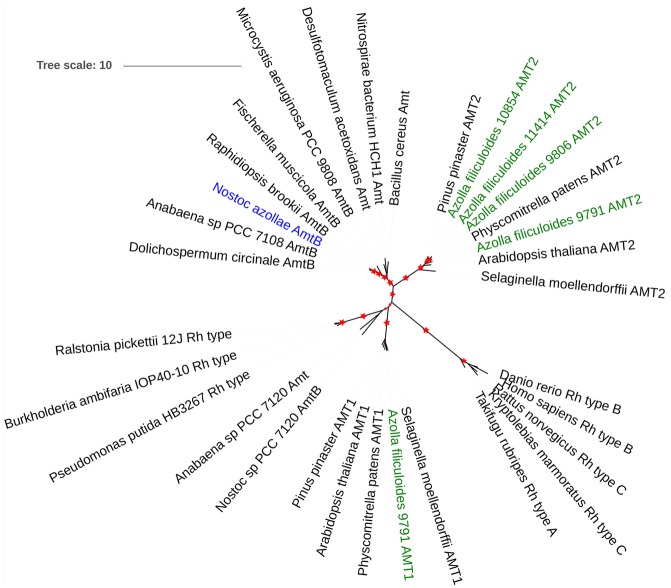
**The origin of ammonium transporters from *A. filiculoides* and *N. azollae*.** Sequences were manually assembled (Supplementary List of AMT Protein Sequences) then aligned with MUSCLE, the phylogenetic tree calculated using PhyML with 100 bootstraps (Dereeper et al., 2008) and then visualized using iTol (Letunic and Bork, 2016), bootstrap values ranging from 70 to 100% are represented by the increasing red star sizes.

Genes affected by +N throughout the day included the nitrite and nitrate reductase genes (Af1086, Af13194, and Af36782, respectively; **Table 1**) and confirm that fern metabolism responded to nitrate in the medium. Similarly increased transcript of the HPP family nitrite transporter (Af1275; Supplementary Table 2_N_responsive) in ferns +N suggested increased nitrite transport to chloroplasts in fern cells; this fern gene is of cyanobacterial origin and was reported absent in mosses (Maeda et al., 2014) In contrast, increased transcript abundance in ferns –N of an AMT2 type ammonium transporter (Af12800; **Table 1**), two phosphate transporters homologous to AtPHT1 and AtPHT3 (Af380, Af1508; Supplementary Table 2_N_responsive), and a Nodule INception (NIN) transcription factor with homology to AtNLP7 (Af7964; Supplementary Table 2_N_responsive) suggest that ferns with active N_2_ fixation regulate P and N transport coordinately as known from the angiosperm symbioses with arbuscular mycorrhiza (Breuillin-Sessoms et al., 2015). Manual assembly of AMT transporter sequences allowed construction of a phylogenetic tree showing that the fern AMT transporters are both of the MEB (AMT2 type in *A. thaliana* which is electroneutral) and AMT (AMT1 type in *A. thaliana*) clades as defined by [Neuhäuser et al., 2009; McDonald and Ward, 2016; **Figure 7**]; AMT2 had increased read counts in ferns with actively N_2_-fixing cyanobacteria (**Table 1**). *N. azollae* AMT clustered with the bacterial AmtB clade, yet the *N. azollae* AmtB that is functional was not in the genome location of closely related strains which was inactivated by a mobile element (Supplementary Figure 6).

### Key Genes of the Vasculature Were Upregulated under –N Growth Conditions

Xylem was a prominent feature of the vasculature curving around the leaf pocket (**Figure 3C**). This specialized vasculature may have as much a structural function as a nutrient and water transport function. In ferns –N compared to +N, transcripts of fern homologs of the xylem cysteine peptidase accumulated (*XCP3*, Af1829) and of the CYTOKININ OXIDASE decreased (Af34233), suggesting active xylem formation (Supplementary Table 2_N_responsive). Further investigation of xylem specific genes revealed homologs of *VND6* and *LBD15* as well as *IRX3* in the *A. filiculoides* sequences. Phloem cells lined the xylem cells for most of the vasculature curving around the leaf pocket (**Figure 3C** and Supplementary Figures 3B,C). To test a possible role of vasculature in the response to differential N supply, full length gene-homologs characteristic for the vasculature were manually assembled then reads mapped to quantify differential accumulation of the mRNA with more accuracy. Transcripts of a fern homolog to *AtGL22* were amongst the most differentially accumulating transcripts (13 × averaged over all time points) under conditions −N compared to +N, GL22 is known from *A. thaliana* to regulate allocation of phloem nutrient contents.

## Discussion

### Light-Driven N_2_ Fixation by *N. azollae* Entirely Supported the High Yields of *Azolla* Biomass

Clones of *A. filiculoides* without *N. azollae* required N-fertilizer for growth and inorganic nitrogen sufficed to reach growth rates nearing those of clones with symbionts (**Figure 3B**). *N. azollae* supplied sufficient N to support high biomass yields with little gain from N-fertilizer (**Figure 1B**). ^15^N_2_-fixation rates for *Azolla* on medium without N-fertilizer peaked at 0.4 mg N g^−1^ DW h^−1^ during midday but averaged 0.17 mg g^−1^ DW h^−1^ over the diel cycle; this corresponded to the N increase in the biomass, reaching 0.33 g m^−2^ d^−1^ in our experiments or 0.15 g^−1^ DW h^−1^. To compare, ^15^N_2_-fixation rates in soybean averaged 0.08 mg g^−1^ DW h^−1^ (Hung et al., 2013). Diel variation of N_2_ fixation in clover (*Medicago truncatula*/*Sinorhizobium meliloti)* was small and entirely dependent upon temperature (Cabeza et al., 2015). The diel variation reported here for *A. filiculoides* was measured under constant temperature and therefore the high N_2_-fixation was diurnal, and depended on light.

*Nostoc azollae* is a filamentous cyanobacterium. Typically N_2_-fixation is sequestered to heterocysts in filamentous cyanobacteria and occurs mostly during the day when, firstly, cells of the filament supply carbon to fuel heterocyst glucose-6-phosphate dehydrogenase (G6PDH) providing reductant NADPH and, secondly, light energy captured by heterocyst photosystem I supplies ATP; G6PDH was required for N_2_-fixation and growth of *Nostoc* sp. in the dark (Gallon, 1981; Summers et al., 1995). *N. azollae* lack glycolysis pathway enzymes and therefore may channel all carbon through G6PDH to generate NADPH required to reduce N_2_; the *N. azollae* genome retained PS1 for ATP production in light (Ran et al., 2010). In contrast, heterotrophic *Sinorhizobium meliloti* fixes N_2_ in the darkness of root nodules and therefore requires carbohydrates to synthesize NADPH as well as ATP: malate provided by the plant provides NADPH via malic enzyme, and is further respired via oxidative phosphorylation specialized for micro-aerobic environments. The malic enzyme gene is required for N_2_ fixation by *Rhizobium meliloti* (Driscoll and Finan, 1993). These differences may underlie the higher N_2_-fixation rates observed here with phototrophic diazotrophic symbionts in *Azolla* compared to heterotrophic diazotrophic symbionts in legumes.

### *Azolla* Yields without N-fertilizer Input Competed Favorably with Established Plant Protein Crops

Nitrate fertilizer at 4 mM increased productivity of *Azolla* by merely 15% (**Figure 2A**) compared to 175 and 132% increase reported when 5 mM NO_3_^−^ was administered to elite breed soybeans 40 and 60 days after planting (Yashima et al., 2003). RNAseq and quantitative RT-PCR revealed that the fern responded to exogenous NO_3_^−^ with the accumulation of transcripts from nitrite and nitrate reductase (**Figures 2A,C** and **Table 1**), and a nitrite transporter (Supplementary Table 2) consistent with previous data of *A. pinnata* showing that labeled NO_3_^−^ is incorporated in roots and then is seen to move slowly to shoots (Ito and Watanabe, 1983). Exogenous NH_4_NO_3_ at 2 mM resulted in a 78.4% decrease in ^15^N_2_-fixation by the symbiont (**Figure 4B**). Similarly, N_2_ fixation decreased by 20–70% in 99 different strains of *Azolla* on 2.86 mM (NH_4_)_2_SO_4_ (Okoronkwo et al., 1989). This may be due to diffusion of external NH_4_ into the leaf pocket: complete inhibition of N_2_ fixation took place with 1 mM NH_4_^+^ but not NO_3_^−^ in the related free-living *Anabaena cylindrica* (Ohmori and Hattori, 1974). Alternatively, the fern may contribute to actively regulate N_2_-fixation in the symbiont by controlling gas exchange via the leaf-pocket pore (Veys et al., 1999) and O_2_ release. Increased O_2_ exposure as evidenced by increased PS2 and hemoglobin expression in ferns +N further suggest this (Supplementary Table 2); NO_3_^−^ or urea from the fern unlikely affect N_2_ fixation in the symbiont directly since genes for import and assimilation of NO_3_^−^ and urea were reported missing in *N. azollae* (Ran et al., 2010). N_2_ fixation by rhizobia in the nodules of legumes is also inhibited by NH_4_^+^: NH_4_SO_4_ at 5 mM rapidly decreased N_2_ fixation in alfalfa roots exposed over a period of 5 days (Cabeza et al., 2015). In plant symbioses with either cyanobacteria or rhizobia, therefore, the N-substrate exchanged when present in the surrounding medium inhibits N_2_-fixation of the bacteria.

In contrast to the large majority of agricultural crops, *Azolla* species grow in shallow surface freshwater. Once the surface of the water was covered by exponential growth, our experimental farming system produced biomass linearly and stably over periods exceeding 130 days (**Figure 1B**). Given a measured N-content of 3.5% w/w biomass DW and 35 t ha^−1^ annual productivity potential without any N-input (**Figures 1**, **2**), *A. filiculoides* has the potential to fix over 1200 kg N ha^−1^ a^−1^ and therefore should be considered as a sustainable plant protein crop in temperate regions. Whilst freshwater aquaculture of macro- and microalgae exceed the yield potential in biomass and protein of the floating *Azolla*, reaching over 100 t ha^−1^ a^−1^ with similar nitrogen content, they have not been studied for their N-fixation capacity, with exception of the cyanobacteria (Neveux et al., 2015). They are generally cultured for wastewater treatment because they require high nutrient loads to reach these productivities. As in the case of *Azolla* more ecological work is needed to be able to reap the benefit of their characteristics and make the most of associated microbiomes for sustainable aquaculture in specific settings (Ramanan et al., 2016). *Azolla* aquaculture appears particularly suited to halt land subsidence in the Dutch lowlands, for example: the dried peat-richt lands now used as grazing and cropping fields emit large amounts of CO_2_ from respiration causing “double trouble” (Erkens et al., 2016). *Azolla* biomass grown on the flooded lowlands has the potential to substitute forage as well as other crops currently grown at lower productivities and be more sustainable stopping N-fertilizer pollution, CO_2_ emmisions and land subsidence. To compare, established forage crops that still require some 150 kg ha^−1^ a^−1^ N-fertilizer to reach 25 t ha^−1^ a^−1^ fix some 600 kg N ha^−1^ a^−1^ in the harvested material (de Visser et al., 2014; Anglade et al., 2015). Modern soybean varieties yielding 5 t beans ha^−1^ with no added fertilizer to the soil fixed up to 300 kg N ha^−1^ per crop (Gelfand and Robertson, 2015). Our experimental system aimed at industrialization and thus was closed and entirely controlled. Promising yields, however, have been reported in an open system of 468 m^2^ in the tropics (Colombia), where *A. filiculoides* yielded 39 t ha^−1^ a^−1^; albeit in this study nitrogen-containing chicken manure was supplied to the fern (Becerra et al., 1990). Feeding *Azolla* biomass to pigs as protein-rich admixtures to soymeal supported growth as well as control soymeal diets (Becerra et al., 1990; Leterme et al., 2009, 2010). Continuous production is particularly suited for on-farm processing as it requires processing units of smaller capacity. *Azolla* ferns efficiently use run-off water from fields and remove both nitrogen and phosphate (Shilton et al., 2012), thereby preventing eutrophication if harvested regularly and contributing to closing the nitrogen and phosphate cycles. If grown as dense mats the ferns may help re-solve iron oxides, while releasing adsorbed phosphate and therefore be an alternative to mining increasingly rare phosphate fertilizer from sediments of shallow freshwater (Cordell et al., 2011). The findings reported here warrant a detailed assessment of potential (agro-) ecosystem benefits and services of *Azolla* farming, including potential threats to protected wetland ecosystems.

### Specific Physiological Adaptations of the Ferns to the Diurnal Cyanobacterial N_2_ Fixation

If hosting cyanobacteria is such an efficient solution to fixing large amounts of N_2_, we wondered why taxa hosting cyanobacterial N_2_ fixation are comparatively rare and what adaptations would be required by the host plants.

Since N_2_ fixation was mostly light driven, the fern host alternated between high N availability during the day and low N availability during the night; low N availability may be restricted to the later part of the night due to buffering by the high NH_4_^+^ concentrations in the *N. azollae* and leaf-pocket fluid (Kaplan and Peters, 1981; Meeks et al., 1987). Consequently, the transcriptional patterns differed in *A. filiculoides* supplemented with NH_4_NO_3_ and without. The morning time point without NH_4_NO_3_ (**Figure 6B**) displayed transcripts highly reminiscent of those in *A. thaliana* when supplied with nitrate after N-starvation (Scheible et al., 2004). The responses to low N availability were therefore conserved between ferns and seed plants. The transcriptional response to low nitrogen availability disappeared during the day with the noon time point having the fewest changes between +N and –N conditions and the highest *N. azollae* N_2_-fixation rates. The magnitude of the fern transcriptional response to N-fertilizer was therefore much lower than that reported for spermatophytes entirely dependent on externally available N (Wang et al., 2003; Vidal et al., 2013). As N supply was short during the night, it is likely that *Azolla* possess a storage system for N which balances availability during the day and the night, this could have been reflected by the prominence of urea transporters in the list of genes accumulating in ferns without N-fertilizer. Night-time accumulation of transcripts encoding enzymes of allantoin metabolism in ferns without N-fertilizer points to allantoin as an intermediate in N storage. To host phototrophic diazotrophic cyanobacteria, therefore, ferns adapted existing responses required to cope with irregular supply of N-fertilizer from the environment that evolved in ancestors common to ferns and seed plants.

### Features That Ensure Metabolic Connectivity between Host and Cyanobacteria

Streptophyte algae and bryophytes were found to regularly associate with N_2_-fixing cyanobacteria as well as rhizobia, suggesting that evolution of land plants, including the fern *Azolla*, happened after mechanisms important for both these interactions evolved (Knack et al., 2015). Recognition of specific cyanobacteria evolved with chloroplast endosymbiosis, but recognition competency may have been lost and subsequently acquired again with a novel mechanism by the common ancestor of vascular plants. Mechanisms employed by the seed plants *Gunnera* and *Blasia* to control development of *N. punctiforme* hormogonia may therefore also occur in *Azolla* ferns (Liaimer et al., 2015). Specific regulators and transporters for the exchange of nutrients with intracellular organelles such as the chloroplasts may not have been recruited for extracellular symbiosis, but instead have evolved from extracellular interactions such as those with arbuscular mycorrhiza, rhizobia or, *Nostoc* species. This would allow differential regulation of the transport processes. The AMT2 and PHT1 family transporters and the NIN transcription factor transcripts seen to accumulate in *Azolla* actively fixing N_2_ could therefore play a role in the interaction with *N. azollae*. Active mobile elements inside *N. azollae* likely contributed to novel regulation of the ammonium transporter expression required for the symbiosis (Supplementary Figure 5) in addition to the metabolic adaptations such as the lacking glycolysis pathway reported earlier (Ran et al., 2010).

The most striking adaptation of the fern to *N. azollae* is the enclosed leaf-pocket organ with its specific pore, leaf-pocket hair cells, and extensive curving xylem-rich vasculature (**Figure 3** and Supplementary Figures 3B,C). The pore structure is likely important for gas exchange; it is lined with specific teat cells possibly having a role in defense against invading organisms and water repulsion (Veys et al., 1999, 2002). The hair cells have been proposed to resemble transfer cells based on EM-cytology detection of dense cytoplasm adjacent to wall ingrowths and abundant ER with ribosomes and mitochondria, and therefore play a role in nutrient exchange (Duckett et al., 1975) consistent with their close contact with the phloem (Supplementary Figure 3B). Finally, the prominent and specific development of xylem cells surrounding the leaf pockets was consistent with a water conducing function; stark re-enforcement of the tracheids furthermore revealed a structural role of the xylem cells not generally described in ferns (**Figure 3C**). Presence of VND6 in *Azolla* suggested that these NAC factors control secondary wall thickening in ferns as in seed plants (Lucas et al., 2013). Stark accumulation of transcripts related to vasculature, and nutrient transport under conditions requiring N_2_ fixation (manually assembled homologs to AtGL22, 13x and AtXCP1, 9x) suggested that vasculature functions are essential for nutrient supply to cyanobacteria, given that the leaf pockets are enclosed structures that do not communicate with surrounding medium. Hosting cyanobacteria in a leaf therefore may require exquisite control over water and nutrient supply as well as structural re-enforcements and a specialized pore structure so as to hold the heavy pool with bacteria in the air whilst at the same time ensuring light and gas supply.

### The Evening Loop of the Clock is Ancestral to Ferns and Seed Plants

Differential diel transcript accumulation was found for 13% of the fern genes. The types of genes which changed and the magnitude of changes observed here were comparable to those changing in diel cycles in spermatophytes (Bläsing et al., 2005) and occurred in all diel patterns (**Figure 6**), hence the combined output of clock and diel signals such as temperature and light results in similar outputs among vascular plants. Among the transcription factors, the CONSTANS-like group of C2C2 transcription factors was enriched in both *A. filiculoides* and *A. thaliana* diurnal transcripts confirming their ancient role in diurnal gene expression. The best blast match to the C2C2 CONSTANS type transcription factor of *Chlamydomonas reinhardtii* Cre06.g278159, is also diurnal [Zones et al., 2015 in Supplementary Table 16]. Nitrogen fertilizer does not influence the diurnal gene expression in *A. thaliana* (Bläsing et al., 2005) and did so only for a very limited number of transcripts in *A. filiculoides* (**Figure 5B**), which may have been a result of diel variability in N-supply in –N ferns rather than less N availability *per se* (**Figure 4**). The similarity also points to an evolutionary origin of the diel patterns before the split of fern and seed plants. While no diel transcriptome analyses from mosses were available for comparison, the analyses of the single celled *C. reinhardtii* show both different and overlapping patterns. Gene expression in the alga was largely diel with ∼80% of genes in diel rhythms (Zones et al., 2015) compared to only 13% in this study and 10–30% depending on criteria in *A. thaliana* (Bläsing et al., 2005); the difference may reflect asymmetric coupling of tissue specific clocks in multicellular organisms (Endo et al., 2014).

*Azolla filiculoides* harbored the classical clock of seed plants (**Figure 5C**). Mosses have been shown to only contain a single loop of the clock comprising the morning but not the evening loop (Holm et al., 2010), while some algae contain various clock components including those of the evening loop (Noordally and Millar, 2014). In *A. thaliana* vasculature enriched transcripts were mostly controlled by the evening loop, whilst the mesophyll cell transcripts were mostly controlled by the morning loop; also the vasculature clock was shown to override that of mesophyll cells (Endo et al., 2014). In the ferns, whilst the evening loop was complete with TOC1, ZTL, GI, and PRR3, some detected with multiple transcripts in the original assembly (Brouwer et al., 2014), the morning loop was less complex compared to *A. thaliana* (**Figure 5C**). Possibly, therefore, the ancestor of vascular plants evolved an evening loop with a function in the vasculature.

## Author Contributions

PB, ABr, VB, AvdW, AT, ABo, and HS planned, designed the research and carried it out; ABr, US, and PB carried out the bioinformatics analyses. Confocal images were from VB. All authors, including G-JR, BU, and AW discussed the results and commented on the manuscript that HS, PB, and ABr drafted. The final version of the manuscript was approved by all authors.

## Conflict of Interest Statement

The authors declare that the research was conducted in the absence of any commercial or financial relationships that could be construed as a potential conflict of interest.

## Footnotes

1 http://mev.tm4.org/
