# Supplementary material for: Metabolic Adaptation, a Specialized Leaf Organ Structure and Vascular Responses to Diurnal N2 Fixation by Nostoc azollae Sustain the Astonishing Productivity of Azolla Ferns without Nitrogen Fertilizer
Source: Front Plant Sci. 2017 Mar 31;8:442. doi: 10.3389/fpls.2017.00442 (PMC5374210; doi:10.3389/fpls.2017.00442)
Supplement: Supplementary file 2 [file Data_Sheet_1.pdf]

Supplementary Material

Metabolic adaptation, a specialized leaf organ structure and vascular responses to diurnal N_2 fixation by *Nostoc azollae* sustain the astonishing productivity of *Azolla* ferns without nitrogen fertilizer

Paul Brouwer, Andrea Bräutigam, Valerie A. Buijs, Anne O. E. Tazelaar, Adrie van der Werf, Urte Schlüter, Gert-Jan Reichart, Anthony Bolger, Björn Usadel, Andreas P. M. Weber and Henriette Schluepmann*

***Correspondence:** Henriette Schluepmann, h.schlupmann@uu.nl

1 Supplementary Data

Supplementary Data files

- The Supplementary_Data_File.pdf:

Supplementary Tables/Figures	Page
**Supplementary Figure 1.** Leaf-lobe arrangement in *A.filiculoides*	2
**Supplementary Figure 2.** Features of the *A. filiculoides* leaves	3
**Supplementary Figure 3.** Cyanobacterial DNA in *A. filiculoides* without and with cyanobacteria	4
**Supplementary Figure 4.** Transcriptional investments in functional categories comparing *A. filiculoides* and *Arabidopsis thaliana*	5
**Supplementary Figure 5.** Diel transcript abundance of major N-trafficking enzymes in *A. filiculoides*	6
**Supplementary Figure 6.** The two loci encoding *AmtB* in the *N. azollae* genome	7
**Supplementary Table 1.** Primers for Q-RT-PCR	8
**Supplementary Table 3.** Pathways enriched in diel transcripts	9
**Supplementary list of AMT protein sequences** (used in Figure 6)	10-12

- Two excell Files:

Supplementary Table 2. RNA seq of diel N-responses in *Azolla filiculoides.*

(including the following sheets: "alldata"; "summary"; "mapping_stats"; "clock genes"; "diel transcripts"; "clusters"; "cluster_ 10"; "N responsive".)

Supplementary Table 4. Functional categories enriched when comparing *A. filculoides* grown without and with 2 mM NH_4NO_3 at the 4 time points during the diel cycle.

2 Supplementary Figures and Tables

2.1 Supplementary Figures

Supplementary Figure 1. Leaf-lobe arrangement in *Azolla filiculoides***.** Leaves are arranged into two rows**.** Each leaf is made of the lower (LL) and upper lobe (UL). The lower lobes resting on the water surface may hold the upper lobes in their aerial position with the dorsal ab-axial surface of the upper lobe facing light. The ventral ad-axial surface of the upper lobe bears the leaf pocket pore (P).

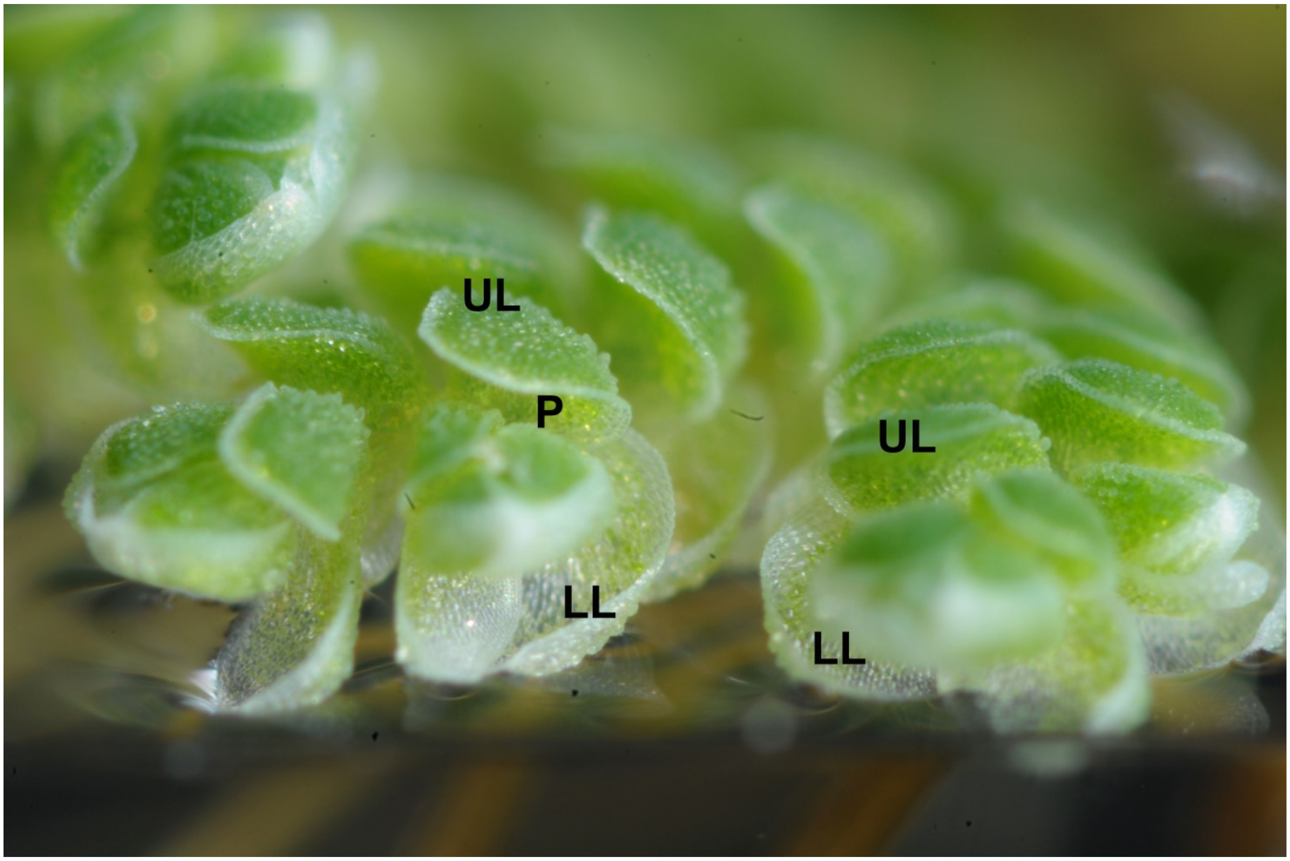

Supplementary Figure 2. Cyanobacterial DNA in *A. filiculoides* without and with cyanobacteria. DNA was extracted from clones of plant 12 without and with cyanobacteria. Subsequently, quantitative PCR was carried out to detect gene abundance from the fern (*TUBULIN* (*AfTUB*), *LEAFY* (*AfLFY*), *AfADENINE PHOSPHORIBOSYLTRANSFERASE* (*AfAPT*) and *FLOWERING LOCUS T* (*AfFT*)) and from the cyanobacteria (nitrogenase (*NaNG*); filamenting temperature-sensitive Z (*NaFtsZ*) and dihydrofolate reductase (*NafolA*). Shown are the average abundances relative to the average abundance of *LFY* from three replicate DNA extractions. Primer sequences for the amplifications are given in supplemental Table S1.

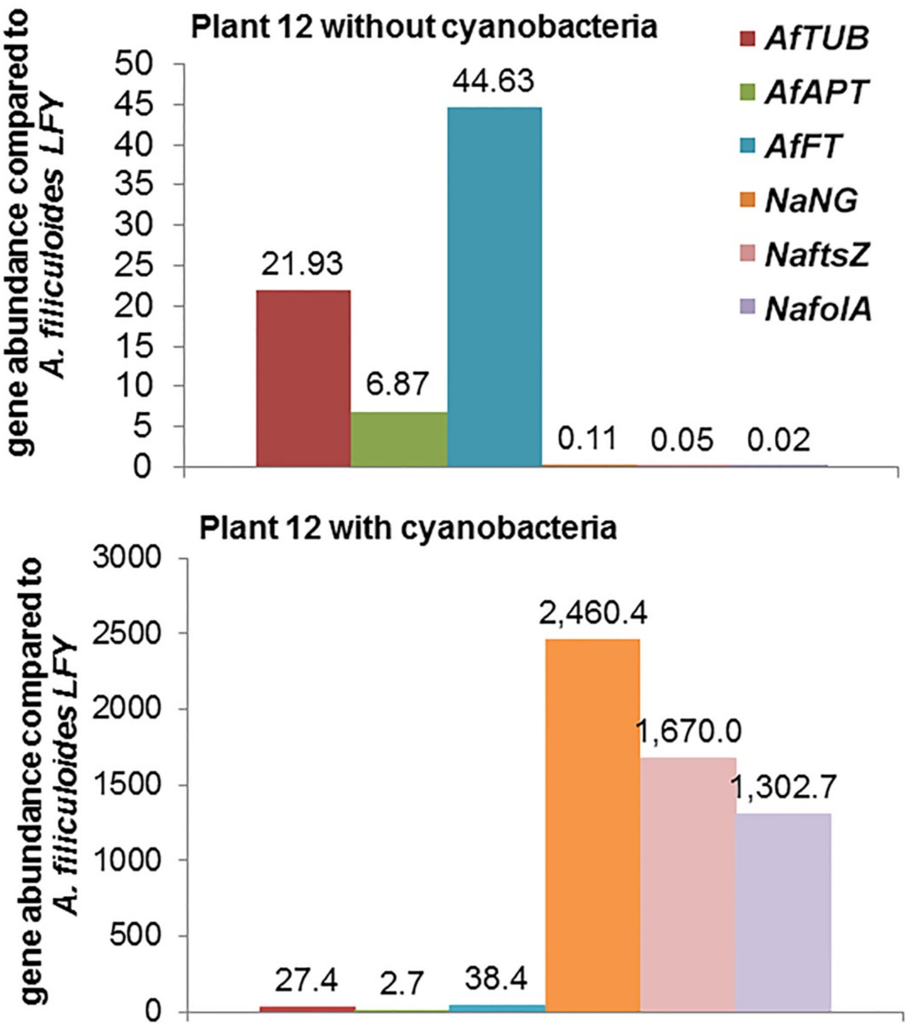

Supplementary Figure 3. Features of the *A. filiculoides* leaves. **(A)**, a single vasculature (v) branches away from the stem, then branches again to each one of the two leaf lobes: the lower leaf lobe without and the upper with leaf pocket. Only the leaf lobe with pocket has a prominent vasculature that curves around the leaf pocket (lp) with its pore (p); r, root; scale bar 200 μm. **(B)** and **(C)**, details of the interface between the leaf pocket and vasculature at the beginning and at the top curvature of the vasculature surrounding the leaf pocket respectively. lh, leaf hair; x, xylem; ph, phloem; t, tracheid cell.

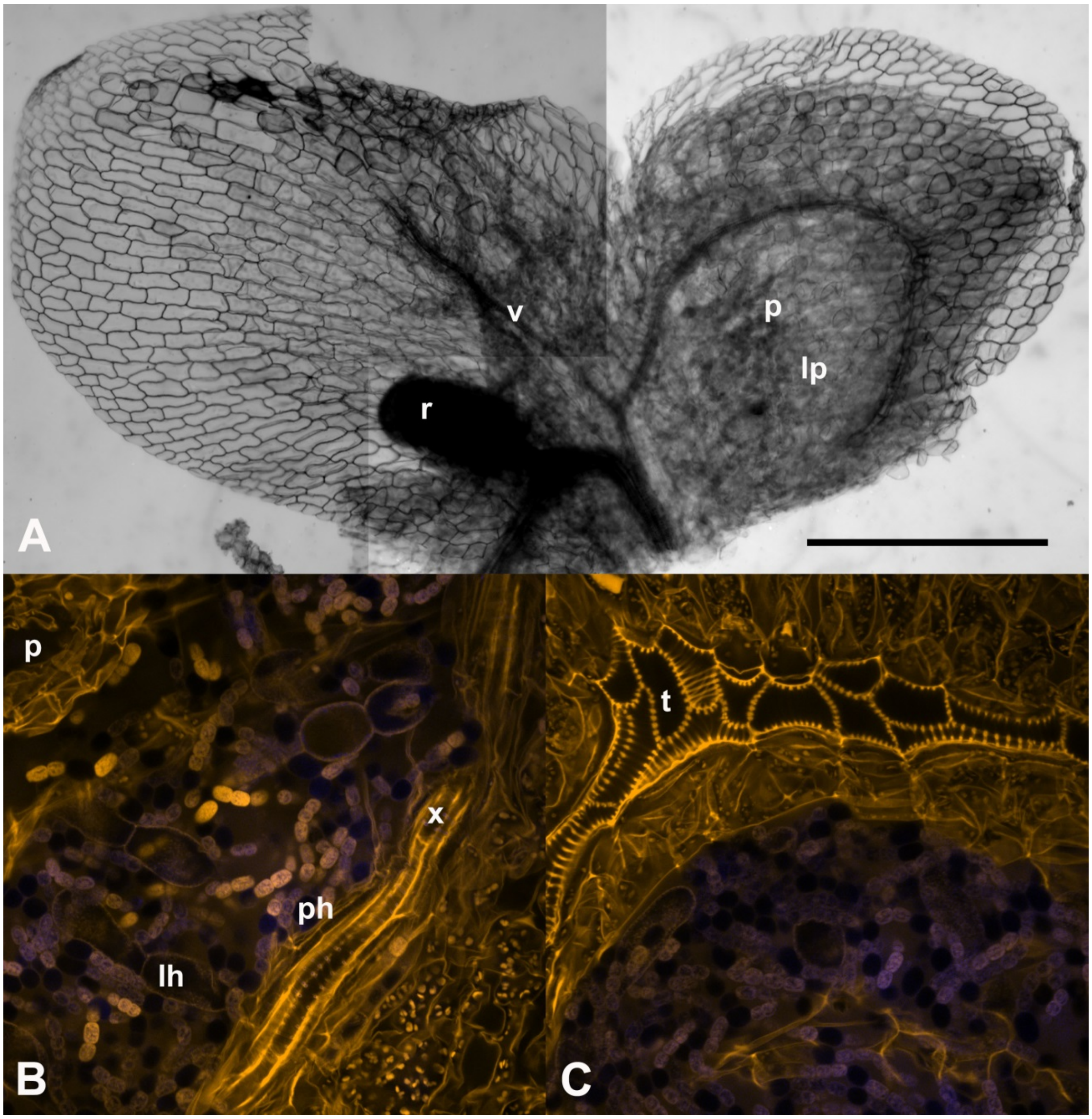

Supplementary Figure 4. Transcriptional investments in functional categories comparing *A. filiculoides* and *Arabidopsis thaliana* seedlings. Investment averages for *A. filiculoides* were for the 3 replicates at each of the time points 2, 8, 14 and 20 h either without or with N-fertilizer. Data of Arabidopsis seedlings was from (Scheible *et al.*, 2004)

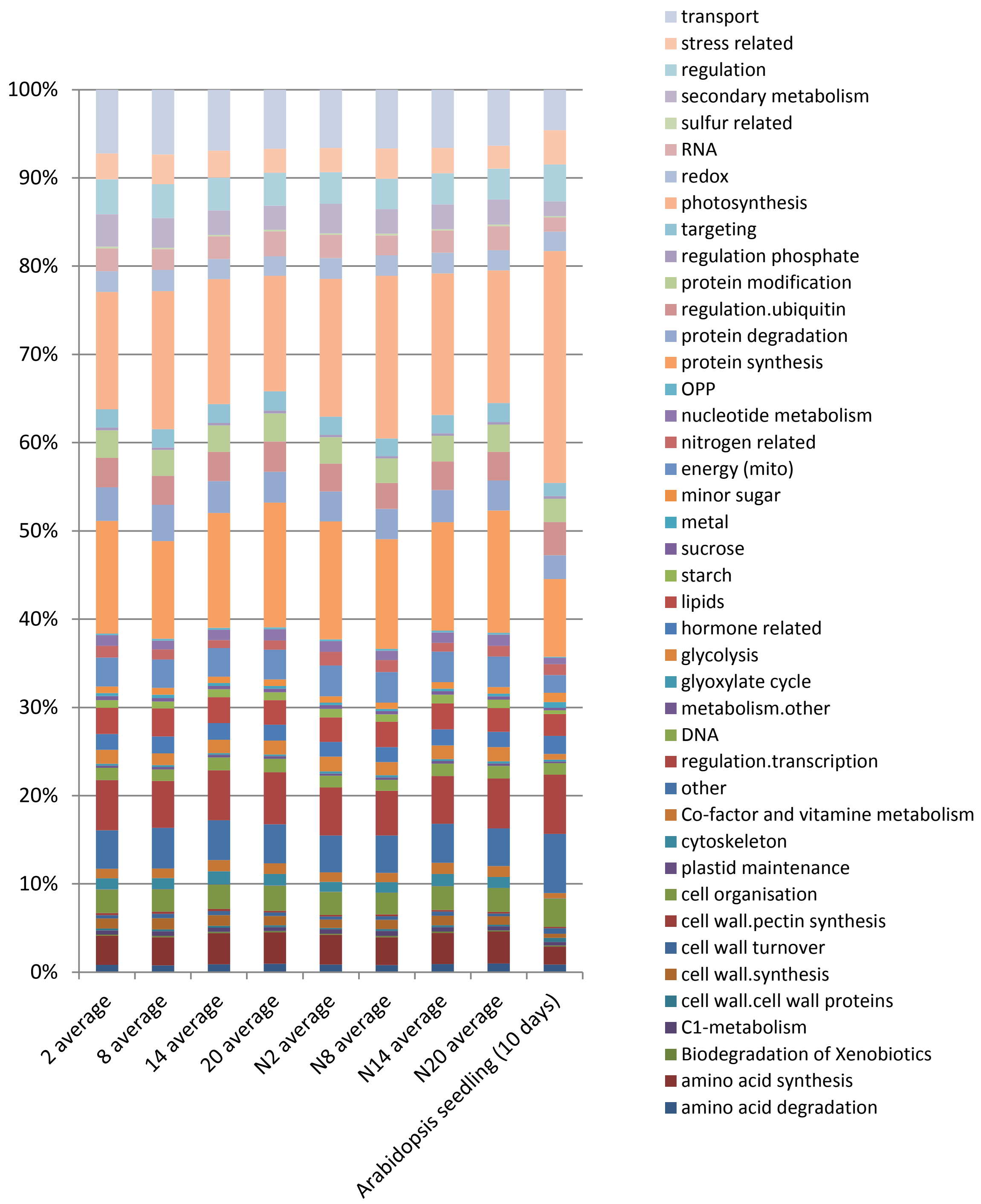

Supplementary Figure 5. Diel transcript accumulation of major N-trafficking enzymes in *A. filiculoides*. Read counts are given as rpm for 2, 8, 14 and 20 h in each box; the numbers are averages from 3 biological replicates for the contig with most reads of ferns on medium without N. AAT, aspartate aminotransferase; ALAT, alanine aminotransferase; AS, asparagine synthase; CPSA, Carbamoyl phosphate synthetase; GAD, glutamic acid decarboxylase; GDH, glutamate dehydrogenase; GFPAT, glucosamine-phosphate n-acetyl transferase; GGAT, glutamate:glyoxylate aminotransferase; GOGAT, glutamate synthase; GPAT, glutamine phosphoribosylpyrophosphate amidotransferase; GS, glutamine synthase; P5CR, pyrroline-5-carboxylate reductase; P5CS, pyrroline-5-carboxylate synthetase. *marks the only transcripts with significantly changed steady states when ferns are grown with N compared to no N.

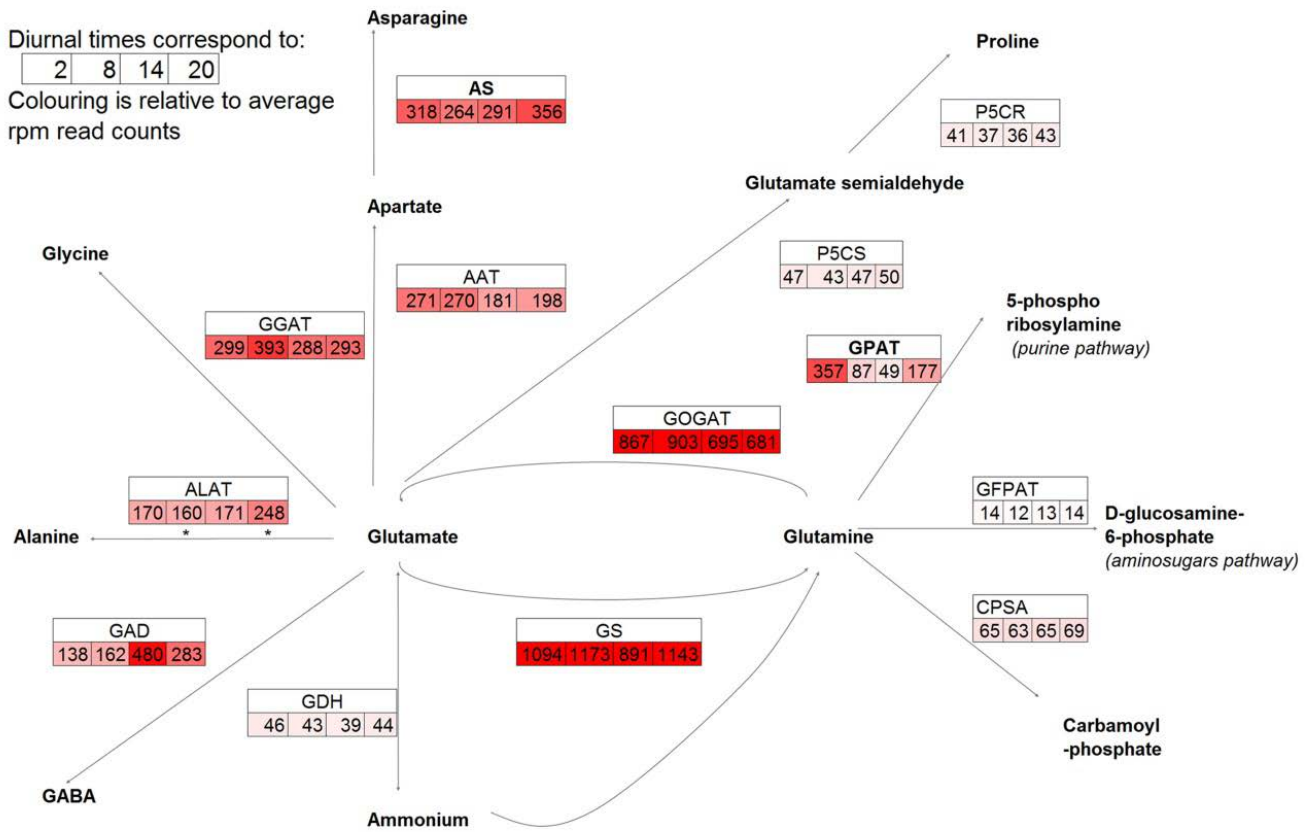

Supplementary Figure 6. The two loci encoding AmtB sequences in the *N. azollae* genome. Annotation of the *N. azollae* genome was visualized with SEED VIEWER (Overbeek *et al.* 2005 Nucleic Acids Res. 7;33(17):5691-702 DOI: 10.1093/nar/gki866). **(A)**, the locus of *N. azollae* similar to the AmtB locus in the closely related *Anabaena variabilis* ATCC 29413 strain contains truncated AmtB fragments flanked by mobile elements (15) ; 1, AmtB; 5, phosphoribosylaminoimidazole carboxylase (EC 4.1.1.21); 9, N-acetylglucosamine-6-phosphate deacetylase (EC 3.1.5.25). **(B)**, a locus entirely distinct from that of the AmtB loci of closely related bacteria contains the functional AmtB of *N. azollae*.

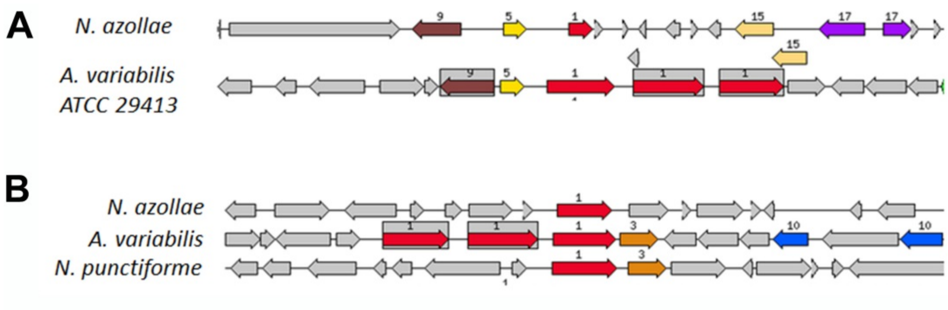

2.2 Supplementary Tables

Supplementary Table 1 Primers used for quantitative Reverse Transcription-PCR on RNA and for quantitative PCR on genomic DNA preparations.

Name	Azolla contig number	Forward primer	Reverse primer
Af_TUB-1	22933	CCTCCGAAAACTCTCCTTCC	GGGGGTGATCTAGCCAAAGT
Af_ACT7-5	3487	TGGGAGAACCACAGGTATTG	TCACGTCCAGCAAGATCAAG
Af_CAS-1	8998	TGGTGGATTCTCAGCAACAG	TCAGCAAACACAGATGACTGC
Af_ATPd-1	1569	AATGGTGGTAATGGCTCTCG	AGGCTTGGTGGTGGATATTG
Af_NR-4	36782	GAAGATCAACCCCTGCAAAG	ATCATCCATCCTCCTCCTTG
Af_APT-1		TAGAGATGCATGTGGGTGCAGT	AAAAGCGGTTTACCACCCAGTT
Af_FT-1		AAGAGATTTGGCAAGCTGGA	TAGCAACCACCAACAGCATC
Af_SOC1-1		ATGGGATCGTAAGGCTTCAAAA	AGCAGAGCACACAGGTCTCAAC
Af_LFY-1		GCGGCAAGAGGAAGAGATAGA	AGTGGATGTGCTCTTGCTGAA
Af_CAL_1		TTTGCATCTTTCGCTCTCTCA	CCAAGCTGCACAATGTAAGGA
Na_NifH-1*		TTCACTCCAAGGCTCAAACC	CGGAAACCGGTCAACATTAC
Na_SecA-1*		AGTATATGGCGCGGTTGAAG	AACAAAGCCTTGAGCACCAC
Na_folA-1*		ATCCTGTGATTCTCGGTCGTAAA	TGCTTCTGCAAAAATTTCTCCTC
Na_FtsZ-1*		TTCGCTATGCTGATGATGTCCTA	GCTCCTTCAATCGAGCATTCTAA

*From the *Nostoc* strain 0780 genome.

Supplementary Table 3. Pathways enriched in diel transcripts determined by Wilcoxon-Rank-Test.

Name	Elements	q-value*
cell wall.modification	24	5.99E-13
cell wall.pectin esterases	46	4.98E-08
major CHO metabolism.degradation.starch	53	3.83E-06
secondary metabolism.phenylpropanoids	74	4.47E-06
amino acid metabolism.synthesis.serine-glycine-cysteine group.glycine	8	7.88E-06
secondary metabolism.wax	36	1.11E-05
PS.lightreaction	115	1.73E-05
major CHO metabolism.synthesis.starch	45	2.48E-05
minor CHO metabolism.trehalose.TPS	12	7.69E-05
amino acid metabolism.synthesis.central amino acid metabolism.alanine	12	7.69E-05
lipid metabolism.Phospholipid synthesis	66	1.25E-04
N-metabolism.nitrate metabolism	3	1.56E-04
minor CHO metabolism.trehalose.TPP	5	1.62E-04
PS.calvin cycle	72	1.67E-04
lipid metabolism.lipid degradation.lysophospholipases	64	2.39E-04
S-assimilation	11	6.71E-04
minor CHO metabolism.raffinose family	9	0.00218605
PS.photorespiration	31	0.0027783
amino acid metabolism.synthesis.aspartate family.threonine	4	0.00281646
secondary metabolism.flavonoids	54	0.00292455
secondary metabolism.isoprenoids	112	0.00462668
lipid metabolism.glycolipid synthesis	10	0.00517521
major CHO metabolism.synthesis.sucrose	17	0.00545819
tetrapyrrole synthesis	57	0.00679658
C1-metabolism	48	0.0082183
N-metabolism.ammonia metabolism	26	0.00980327

***** q-value was corrected for multiple hypothesis testing by Benjamini Hochberg; only significantly enriched pathways are shown.

3 Supplementary list of AMT Protein sequences

>*Azolla_filiculoides*_9791_AMT2
MATSVPDWLDKGDNAWQLVAATIVGMQSMPGLVILYGSIVKKKWAVNSAFMALYAFAATLLCWVTWAYKMSFGEKLLPIWGKAGPALGI
DYLIGNAHIPASVHLDSSGAVETAEIAPAFPMATLVYFQFVFAAITVIIVAGSLLGRMNFKAWMLFVPLWLTFSYTVSAFSLWGGGFLFQWGV
GPRLKKDKDNFPPNNILMTLAGAGLLWLGWSGFNGGSALAANVSASIAILNTHVCTATSLLVWISLDIIFLGKPSVIGAVQGMTTGLVCITPAA
GLVQGWAAIIMGVLSGSIPWFTMMVLHKKLHILQHVDDTLGVLHTHAVAGILGGILTGLFAHPTLCSFFAPVKGARGSIYGGQGRDQVGRQL
AGALFVVAWNVVVTSIICLGIKMVMPLRMSNNELLMGDDAAHGEAAYALWGADDKPGQVFTSVEYDNNASYHNNAQYIASTATIQL
>*Azolla_filiculoides*_10854_AMT2
MSGGVPSAYNNTGGVLPDWLNKGDNAWQMTAATLVGLQSMPGLVILYGSIVKKKWAVNSAFMALYAFAAVLICWVIWAYKMAFGEKLLP
FWGKAGPALGQHYLVSQAALPETAHYHKDGTLETAEITPFYPMATMVYFQAEFAAITVILIAGSLLGRMNIIAWMIFVPLWLTFSYTVGAFSL
WGGGFLFQWGVIDYSGGYVIHVSAGTAGFVAAYWVGPRLTKDRERFPPNNVLLTLAGAGLLWLGWAGFNGGDPYSANIDSSIAVLNTNVC
AAASLLVWTSLDVIFFKKPSVIGAVQGMITGLVCITPAAGVVEGWAAIIMGILAGSVPWFTMMVVHKKSKILQKVDDTLGVFHTHAVAGTLG
GLLVGLFAEPTLSSYFLPVQNSRGGFYGGKAGGEQFGKQLVGALFIIGWNVVSTTIILLLIKMIVPLRMTDDELLIGDDAAHGEEAYALWGDG
EKFDDTQHSHIDHPLTTFSPYYGRGTVEL
>*Azolla_filiculoides*_9806_AMT2
MALPMAYANNTSSVPDWLNKADNAWQLTAATLVGLQSVPGLVILYGSIVKKKWAVNSAFMALYAFAATLLCWVTWAYKMAFGDRLLPM
WGKAGPALSQDYLVGRAYLPATEIRGHISEGVGESNTPIIHPFFPMAAMVYYQFAFAALTIIILAGSLLGRMNIKAWMLFVPLWVTFSYTVGAF
SLWGGGFLFQWGVLDYSGGYVIHVSSGVAGLVAAFWVGPRLTKDRERFPPNNILLTLVGAGLLWLGWAGFNGGSGFSADIDSSLAVLNTHV
CTATSLLVWAALDVLIFGKPSVIGAVQGMTTGLVCITPAAGLVQGWAAIVMGLLAGSVPWFTMMVVHKKSRILQHVDDTLAVFHTHAVAGI
LGGLMTGLLADPILCSYFRPGVKSGGSFYGGQGKDQIGRQLVGALFIIGWNVVTTSLICLAIKLVVPLRMSVEELSTGDDAAHGEEAYALWGD
GEKFNDNRFNVTSSNIPMSNLQDAEHGIIATHYNLPKESLTTSSTVHI
>*Azolla_filiculoides*_11414_AMT2
MALPMAYANNTSSVPDWLNKADNAWQLTAATLVGLQSVPGLVILYGSIVKKKWAVNSAFMALYAFAATLLCWVTWAYKMAFGDRLLPM
WGKAGPALSQDYLVGRAYLPATEIRGHISEGVGESNTPIIHPFFPMAAMVYYQFAFAALTIIILAGSLLGRMNIKAWMLFVPLWVTFSYTVGAF
SLWGGGFLFQWGVLDYSGGYVIHVSSGVAGLVAAFWVGPRLTKDRERFPPNNILLTLVGAGLLWLGWAGFNGGSGFSADIDSSLAVLNTHV
CTATSLLVWAALDVLIFGKPSVIGAVQGMTTGLVCITPAAGLVQGWAAIVMGLLAGSVPWFTMMVVHKKSRILQHVDDTLAVFHTHAVAGI
LGGLMTGLLADPILCSYFRPGVKSGGSFYGGQGKDQIGRQLVGALFIIGWNVVTTSLICLAIKLVVPLRMSVEELSTGDDAAHGEEAYALWGD
GEKFNDNRFNVTSSNIPMSNLQDAEHGIIATHYNLPKESLTTSSTVHI
>*Azolla_filiculoides*_9791_AMT1
MATATWNTSCLASSTIDPSVCAQFDVVNAHLIATTLAINNTYLLFSAYLVFAMQLGFAMLCAGSVRAKNTMNIMLTNVLDAASGGLSYYLFG
FAFAFGRKSDGHNNGFIGRHFFGLKQIPDELNGFDYSFFLYQWAFAIAAAGITSGSIAERTQFTAYLVYSCFLTGFVYPVVSHWLWSVDGWLS
ATNPSLLFGSGAIDFAGSGVVHMVGGIAGLWGAFIEGPRMGRFDADGQPQRLKGHSATLVVLGSFLLWFGWYGFNPGSFLVILASPYDTFKG
NWSGVGRTAVTTTIAGSTAALTTLFGKRALGGHWNVLDVCNGLLGGFAAITAGCSVVDPWASIVCGFVSAWVLIGLNLLAEKLKFDDPLEA
AQLHGGCGAWGLIFTGLFANENYVRQVYGRTGDNVQYGLFMGGGGRLLGAQIVEILSIIGWVTATMAPLFFILHKLNLLRISPKDEFVGMDPT
RHGGHAYYHHDEDPGLSPYKNMTQLKELKTGAPPPEVLVG
>*Arabidopsis_thaliana*_AMT1;2
MDTATTTCSAVDLSALLSSSSNSTSSLAAATFLCSQISNISNKLSDTTYAVDNTYLLFSAYLVFAMQLGFAMLCAGSVRAKNTMNIMLTNVLD
AAAGAISYYLFGFAFAFGTPSNGFIGRHHSFFALSSYPERPGSDFSFFLYQWAFAIAAAGITSGSIAERTQFVAYLIYSTFLTGFVYPTVSHWFWS
SDGWASASRSDNNLLFGSGAIDFAGSGVVHMVGGIAGLCGALVEGPRIGRFDRSGRSVALRGHSASLVVLGTFLLWFGWYGFNPGSFLTILK
GYDKSRPYYGQWSAVGRTAVTTTLSGCTAALTTLFSKRLLAGHWNVIDVCNGLLGGFAAITSGCAVVEPWAAIVCGFVASWVLIGFNLLAK
KLKYDDPLEAAQLHGGCGAWGLIFTGLFARKEYVNEIYSGDRPYGLFMGGGGKLLAAQIVQIIVIVGWVTVTMGPLFYGLHKMNLLRISAED
EMAGMDMTRHGGFAYAYNDEDDVSTKPWGHFAGRVEPTSRSSTPTPTLTV
>*Arabidopsis_thaliana*_AMT2
MAGAYDPSLPEVPEWLNKGDNAWQLTAATLVGLQSMPGLVILYASIVKKKWAVNSAFMALYAFAAVLLCWVLLCYKMAFGEELLPFWGK
GGPAFDQGYLKGQAKIPNSNVAAPYFPMATLVYFQFTFAAITTILVAGSVLGRMNIKAWMAFVPLWLIFSYTVGAYSIWGGGFLYQWGVIDY
SGGYVIHLSSGVAGFVAAYWVGPRPKADRERFPPNNVLLMLAGAGLLWMGWSGFNGGAPYAANLTSSIAVLNTNLSAATSLLVWTTLDVIF
FGKPSVIGAIQGMVTGLAGVTPGAGLIQTWAAIIIGVVSGTAPWASMMIIHKKSALLQKVDDTLAVFYTHAVAGLLGGIMTGLFAHPDLCVLV
LPLPATRGAFYGGNGGKQLLKQLAGAAFIAVWNVVSTTIILLAIRVFIPLRMAEEELGIGDDAAHGEEAYALWGDGEKFDATRHVQQFERDQ
EAAHPSYVHGARGVTIVL
>*Physcomitrella_patens*_AMT1;1
MERIQGFLGAVSNATGLSIFLCDKLDNIDGRLGYTKLAVDNTYLLFSAYLVFAMQLGFAMLCAGSVRAKNTMNIMLTNVLDAACGGISYYVF
GFAFAFGLGGKTNGFIGHFNWGLNGFPNGTFDYSFFLFQWAFAIAAAGITSGSIAERTQFVAYLVYSSFLTGFVYPIVSHWLWSADGWLSASK
TVGPGGLLFGSGAIDFAGSGVVHMVGGVAGFWGALIEGPRIGRFDKSGNSTNFRGHSATLVVLGTFLLWFGWYGFNPGSFLTILQPYEGVKG
HWSGVGRTAVTTTLAGCTAAVTTLFGKRFLDGHWNVLDVCNGLLGGFAAITASCSVVAPWASILCGFGSAWVLIGLNKLAARLHFDDPLEA
AQLHGGCGAWGLLFVGLFAEKNYVNQVYQTTFDSPYGLFMGGGGKLLAAQIIEIISIAAWVTVTMGPLFYGLHKFRLLRITPQDEIAGMDVT
RHGGTAYIHHDNSEHHLQMHTINGQKRTDNDQMPI
>Pinus_pinaster_AMT1;1
MSNFDYSCTAPELAMLQSLLNGTAGDFFCNKLQAVSDRLSATSRAVDSTYLLFSAFLVFSMQLGFAMLCAGSVRAKNTVNIMLTNVLDAAA
GGIFYYLFGFAFAFGRGKHSNGFIGHYFFGLTEVPNQEYQYDYAYFLYQWAFAIAAAGITSGSIAERTQFVAYLIYSSILTGFVYPVVSHWVWS
PDGWMSASNASGLLFGSGVYDFAGSGVVHMVGGIAGFWGALIEGPRIGRYDKEGKPNGIRGHSATLVVLGFPSLWFGWYGFNPGSFAKILVP
YGAGGGSYVDGQWTAIGRTAVTTTLAGCSAALTTLFGRRLLTGHWNVLDVCNGLLGGFAAITGGCSIVDPWAAILCGFVSAWVLIGFNILAG
KMKYDDPLEAAQLHGGCGAWGIIFTALFAKEEYILQAYGLLPNGKSSRPSGLFMGGDGRLLAAHVIQIIVTTGWVTATMAPVFYVLHRFKLL
RVSAADEMAGMDVTRHGGGAYVYHDSDDDKHIHPGGFMMKASRNMTEFPHNNNYRNGAEDNDDDTL
>*Selaginella_moellendorffii*_AMT1;1
MALAAVASIRCSSQDLLSFGLAANASSALCSKLDFVADRLYATQLAVDNSYLLFSAYLVFAMQLGFAMLCAGSVRAKNTMNIMLTNVLDAA
CGGISYYLFGFAFAFGTGGTTNGFIGRYFFALSEIPDSSAGFDYSFFLFQWAFAIAAAGITSGSIAERTQFVAYLIYSTFLSGFVYPIVSHWAWSV
DGWASASKPSGRLFGSGAIDFAGSGVVHLVGGLAGFWAAQIEGPRIGRFDKGGAATLVLKGHSASLVVLGTFLLWFGWYGFNPGSFVTILSP

YGAGSFTGNWTGVGRTAVTTTLAGCSAAITTLFGRRLLTGHWAVVDVCNGLLGGFAAITAGCSVVDPWASLICGFVSAWVLIGLNLLAERFH
YDDPLEAAQLHGGCGTWGLIFTALFAKEEHVLNVYGRTSTPYGLFLGGGGRLLAAQIVYILAIVGWVTVTMGPLFWMLHRLNLLRISPEDEV
AGMDLTRHGGMAYYHQDGSHHDGHKFQLHNLSKHGGGNAPGMVHDAASPL
>*Selaginella_moellendorffii*_AMT2;1
MKCSYLSSSSTPCWMDKGSNAWMMVAAILAGLATMPGLLLLYSGIARKKWAVNTAFLTLYAFTASLICWVTICHNLAFGSHLLPFWGTPGP
VLYTKFLLSRSQHPATHQDLDFPSATMVAFQFGFAANSVAIVSSAVSARITFQAWAVFVPLWLIFSYTVGASSIWSGGFFSRWGVLDFAGGYV
VHLSAGVSGAVLAHWVGPRHPVDRARYPPNNVMLVLAGAGLVWLGWIGFAGGSAFLSPQQASLAVVNTNIAAATSLLVWTSLDVFYHGQP
SVLGAVQGLMTGLVAISPAAGLVEGWASMCIGLCSGSLPWLTRLCHQKTTAKFCEEVDDTAGAVHTHGIAALIGVLLTGFFAHPRLTAMVSP
VSGSEGVIFGNFQLVLKQLAAALLVILWNVAVTTLICVVVKRLMKLRMSDDQLRIGDDAVHGEEAYAVWEDGEKTTL
*>Physcomitrella_patens*_AMT2;7
MADALPTVPIAYNQTGGVTPAWLNKGDNAWQLTASTLVGVQSVPALVILYGSIVKKKWAVNSAFMAFYAFAAVWLCWVGWAYKMSFGE
KLIPIWGKAGTTLSYKYLLSQAELPSTAHYHKNGDIETYALTPFFPMASLVYFQFVFAAITLVLLAGSVLGRMSFRAWMLFVPLWLTCSYTVG
AFSLWGGGFLWQWGVIDYAGGFVIHLSSGIGGFVAAYWVGPRLTKDRERFPPNNVLLMLAGAGLLWMGWAGFNGGAALSANLIASIAVMN
TNVCAATSLLVWTCLDVLIFGKPSVIGAVQGMITGLVVITPAAGLVQGWAALVMGVFAGSVPWFTMMVVHKRSSILQRVDDTLGVFHTHAV
AGLVGGLLVGCFAEPTLCDYFLPVLGERGAFYGGVGGKQLGKQIVGALFITAWNVVMTSIILNVIKLVMPLRMTDEHLLVGDDAEHGEEAY
ALWGDGEMFDMSKHGALTSNLSTLSSNDFGSEKSRPTITL
>*Pinus_pinaster*_AMT2;1
MATYLPTAYQNGTTSPDWLNKGDNAWQMVAATLVGMQSMPGLVILYGSIVKKKWAVNSAFMALYAFSAVMICWVTWAYKMAFGHKLLP
LWGKAGPALGQKYLIGAADLPASQHNHHNGTLETAMIAPFFPMATMVFFQFSFAAITLILLAGSVLGRMNIKAWMAFVPLWLTFSYTVGAFS
LWGGGFLFQWGVIDYSGGYVIHVSSGISGFTAAYWVGPRLTKDRERFPPNNVLLMLAGAGLLWMGWSGFNGGDPYSANIDSSMAVLNTNIC
AATSLLVWTCLDVIFFGKPSVIGAVQGMITGLVCITPAAGVVQGWAAIAMGVLSGIIPWFTMMVLHKRSTLLQKVDDTLGVFHTHAVAGVLG
GALTGLFAEPTLCSLFLPVTNSKGAFYRGPGGVQFLKQLAGACFIIGWNVVATSIILLVISLVIPLRMTDEQLLIGDDAIHGEEAYALWGDGEKY
DNTKHGWYDDTTAGDGRGQGARGVTIEL
>*Nostoc_azollae*_AmtB
MLKKVLTIGVLTLFLLTFPLMGNALAQGRSTPPDPDTGDTAFMLISSALVLLMTPGLAFFYGGFVRSRNILNTLMMSFVLMAIIGVTWVLWGY
SLSFAPGVPFIGGLEWLGLNGVGLETTGYLEDSAPAEVVSYAGTIPHQAFMIYQAMFAIITPALISGAIAERMSFRAYCLFVLLWSTFIYTPLAH
MVWAKGGFLGLYGGLGALDFAGGTVVHISSGVSALVAAIVLGPRKSHPDRLSPPHNVPFILLGAGLLWFGWFGFNAGSALSAGSIATAAFVA
TNTAAAAGTLMWLILEATLRGKPTAVGAATGAVAGLVGITPAAGFVTPLAAILIGFITAFLCFYAVSFKHKLNVDDALDTFPVHGVGGTLGAI
LTSIFATTEVNSGGKDGVLRGNFREFFVELAAIAIAYIIAGTGTWIILKIIDATIGLRVKEEAENQGLDIHEHGEEGYNSEFGDRINV
> *Nostoc_sp*_PCC_7120_AmtB
MAINTGKNKLMNRHIRPWQRLLVLAIGSMVFAVFAPTIVQAVDTPTLESLSETTIKLQISIDTTWVLLSGFLVFFMQTGFAMLEAGLVRQRSVV
NTLLENFIDAAVTVLAWWAVGFGIAFGTSAGGLFGIDTFFLSQLPGADGSYPLGAPGSTAAINTYTLFFFQFAFAATASTITTGSMAGRTDFIGD
LIYSAIMGAISYPIIVHWAWNSNGWLGKLSYHDFAGGSIVHTVGGWTALVGAYLLGPRPDRPPWGKLPPAHNLALATLGTMILWFGWYGFN
PGSTLGTANPGLIGLVTINTTLAAGAGALAALIFLYVRTGKWDLVYCLNGSLAGLVAITAPCAYVAPWASVLIGLTGGIAVVLGVSLIESLHID
DPVGAFSVHGISGMMGTLSIGFLGQEELTLNQKAGLLLGGGFDLLGIQMLGIVAITVFTVAFAFLMYGGLKAMGHLRVNAEADRIGIDTYEHG
ASVWPDVYSVEELSKPQEHTKISENKTLEGE
>*Anabaena*_sp_PCC_7120_Amt
MYQQKSRTRNRRFSTRNYAKSRQSNSQIQIFNLVKKLSPSWQACIPLACLIVLGWSYVAVAQAPAAGPTTAELKVALDTLWVAIAAFLVFFM
NAGFGMLETGFCRQKNAVNVLAKNLIVFALATVAFWAIGFGLMFGDGNDFIGFNGLFLSGVDNSPATGDAYKGVFSALSWAGVPLAAKFLF
QLVFAGTAATIVSGAVAERIKFVDFLIFSLLLVGIAYPITGHWIWGAGWLAKAGFWDFAGSTVVHSVGGWAALMGAAFLGPRIGKYQDKQIV
ALPGHNMSIATLGCLILWLGWFGFNPGSVMAADPNAITHIALTTNMAGAVGGIAATATAWLYLGKPDLSMIINGILAGLVGITASCAYVSIPSS
IIIGLIAGVIVVFSVTFFDKLGIDDPVGATSVHLVCGVWGTLAVGLWSVGPGVYSWYGEGLGPTKGLFAGGGLGQLITQFLGAAAVGGMTVL
VSSIFWVVLKATLGIRVTREEELEGLDIGEHGMEAYSGFLKEASPGGFAEGKTSDGY
>*Anabaena_sp*_PCC_7108_AmtB
MLKKFVMIGVLTGFLLAFPLLGSALAQGTATPPAPDTGDTAFMLISSALVMLMTPGLAFFYGGFVRSRNILNTLMMSFVLMAIVGVTWVLWG
YSLSFAPGLPFIGGLQWLGLNGVGLETTGYLEGSAPAEVVSYAGTIPHQAYMIYQAMFAIITPALISGAIAERMSFRAYCLFVLLWSTFIYTPLA
HMVWAKGGFLSLYGGLGALDFAGGTVVHISSGVSALVAAIVLGPRKNHPDRLSPPHNVPFILLGAGLLWFGWFGFNAGSALSAGSVATVAF
VATNTSAAAGALMWLILEATLRGKPTAVGAATGAVAGLVGITPAAGFVTPLSAILIGLMTALVCFYAVSFKHKLNIDDALDTYPVHGVGGTL
GAILTAFFATTEVNSGGKEGVLRGNFGELFVELGAIAIAYIIAAVGTWLILKFIDSTIGLRVKEETENQGLDINEHGEEGYNSEFGDRINLS
>*Dolichospermum_circinale*_AmtB
MLKKVVITGCLTLLMLGGLLTGNAWAAESTAAPPPDTGDTTFMLISSALVLLMTPGLAFFYGGFVRSRNILNTLMMSFVLMAIVGVTWVLW
GYSLSFAPGLPFIGGLQWLGLNGVGLETTGYLQGSAPAEVVSYAPTIPHQAFMIYQAMFAIITPALISGAIAERMSFRAYCLFVVLWSTFIYTPL
AHMVWAKGGFLGLYGGIGALDFAGGTVVHISSAVSALVAAIVLGPRKNYPDRLSPPHNVPFILLGAGLLWFGWFGFNAGSALSAGTVATVAF
VATNTSAAAGSLMWLILEATLRGKPTAVGAVTGAVAGLVGITPGAGFVTPLAAILIGFITSFVCFYAVSFKHKLQVDDALDTYPVHGVGGTIG
AILTAFFATTEVNSGGKDGVLRGNLSELFVELVAIALAYIIAGVGTWIILKIIAATVGLRVPDQTENQGLDIHEHGEEGYNSEFADRISNK
>*Raphidiopsis_brookii*_AmtB
MLRKFLVISGLIVLLLTFPFAGNAVAQTNTPAPDTGDTTFVLMSSALVLLMTPGLAFFYGGFVRSRNILNTLMMSFVLMAIVGVTWVLWGYS
LSFAPGLPFIGGLQWLGLNGVGLETTGYLKGSVPQEVLSYASTIPHQAYMIYQAMFAIITPALISGAIAERMSFRAYCVFVLMWSTFIYTPLAHA
VWAKGGFLGLYGGLGALDFAGGTVVHISSGVSALVAAIVLGPRKNHPDRLTPPHNVPFILLGAGLLWFGWFGFNAGSALSAGTIATVAFVAT
NTSAAAGALMWLILETNLRGKPTAVGAATGAVAGLVGITPAAGFVTPLAAILIGFITSFVCFYAVSLKHKLNVDDALDTYPVHGVGGTIGAVL
TAIFATTEVNSGGKDGLLRGNFGELFVELGAIAVAYVIAAVGTWLILKFISATIGLRVQEEAEDQGLDVYEHGEEGYNSEFSDRINI
>*Fischerella_muscicola*_AmtB
MLKKVVMIGAIALVLLAGPLIGNALAAPVDVNAAISNAQTAADTAFMLISAALVLLMTPGLAFFYGGFVRSRNVLNTLMMSFVLMGVVGVT
WILWGYSLAFAPGNPFIGGLQWLGLNGVGTELTDYLKGSNPPEILSYAPTIPHLAFMIYQAMFAIITPALISGAIAERMSFTAYSLFVLLWSTFV
YSPLAHMVWGKGGFIGLAGGLGALDFAGGTVVHISSGVSALVAAIVLGPRKTYPDRLSPPHNVPFILLGAGLLWFGWFGFNAGSALASGSLA
TIAFVNTNTSAAAAALTWLILEKVLRGKPTAVGAATGAVAGLVGITPAAGFVTPLAAILIGSITTFVCFYAVSFKHKIQIDDALDTYPVHGVGG
TVGAILTAVFATTAVNSAGKNGLLYGNPRELLVELAAIAIAYVVAGVGTFVILKLIDATVGLRVKEVAEMQGMDISEHGEEGYNEEFGDRISV
SDK
>*Microcystis_aeruginosa*_PCC_9808_AmtB
MLLLGLGFNFGVAPVFAETVAAAPPNPISAGDTAWMLISSALVLLMTPGLAFFYGGLVRSRNVLNTMMMSLVAMGVIGVTWVLWGYSLAF
DVTPGKSGFGEGIEAFIGGLDWIFLNGVTAAAPDNIGYAPTVPHQVFMVYQMMFAIITPALISGAIVERVNFKAYFWFLLLWSTFIYSPLAHWV
WGKGWLAATGALDFAGGTVVHVSSGISALVAAWVIGPRRSFGTQTYAPHNVPFVLLGIGLLWFGWFGFNGGSALSSSSLATTAFVNTTIAAS

AGGLTWMFIEWILRGKPTAIGIASGFLGGLVGITPAAGYVLPIGAILIGSITALSCFFAVSLRAKLRFDDSLDTYPVHGVGGTIGAILTGVFATKS
VNSFGNDGLLFGNPGLVWTQFVGVAATYIFAAVGTFVILKVLSLFMDLRVKPNTEAEGLDVPQHGEEAYGQEFEFGSSFSYQENPPSTNPREN
Y
>*Desulfotomaculum_acetoxidans*_Amt
MLKNLWRLVLVTLSMLLLPGLAWAEDAVPPAINAGDTAFVLMSAALVLLMTPGLALFYGGMVREKNVLSVIMQSFIVIGLVSVQWVLFGYS
AAFGPDIKHIIGSMDWAGLANVGQDPNTDYAGTVPHLAFMSFQLMFAIITAALITGSFAERMRFPAFLIFTLLWTSLVYDPLAHWVWGVDGW
LRNLGALDFAGGTVVHISSGVSGLVAAIVLGRRKGYGTEPMIPHHLPMTVLGASLLWFGWFGFNAGSAVSANGLACSAFVVTNTAAAAAAL
SWVAVEWMRHGKPTVLGAASGCVAGLVAITPASGFVSPLAAIVIGLVAGVLCYLAVSVLKTKLGYDDSLDAFGVHGLGGTWGAVATGLFA
SKAVNSAGNDGLFYGNAAQLVTQIETVAVTWLFAGLATFIILKVVGLFCKLRVDADDEEAGLDITQHGEEAYAANVSSGTLFRDTMA
>*Nitrospirae_bacterium*_HCH-1_Amt
MRYIFTLLFMLIATTAYSDTPAGVDKGDTAWILVSAAMVMFMTPALAMFYGGMVRRKNVLSIIMQSFAAIALVSLQWILFGYSLAFGPDFHGI
IGGLDWAGLSSVTLEPNPDYAPTVPHMAFMIYQAMFAAITPALISGAFAERMRFSAFIAFTFIWALVVYTPVAHWVWGKGGWMSQMGVLDF
AGGIVVHVTSGFSALAAALYLGKRKGFPHDLMPPHNLPLTVIGTGILWFGWFGFNGGSALSSGQLSTLAFVTSHTAAVSAMCVWITLEWMLH
GRPTMFGAATASIAGLATVTPAAGFITPMAALAIGCVAGVVCYFALNLKPRFGYDDSLDAFGVHGVGGAIGTICLGLFASKAINANGADGLFY
GNAKQLIIQLAAVLIVAVFSFVMTIVIFKIISLFTSIRVNTEDEVEGLDTTQHGESGYYM
>*Bacillus_cereus*_Amt
MNTGDTVFMFVTTVMVMLMTPGLALFYGGMVRSKNVLSTTMHSYSAMAIVSIQWIVIGYSLSFGPDWHGLIGTLDWFGLNGVTYAPNPDYS
STIPHNLFMMFQLMFAILTPALISGAFAERMRFSAFLIFILLWTTIVYNPVAHWVWGVGGWLRELGALDFAGGNVVHITSGVAGLVLAIFLGK
RKNINGSSPHHLPFTMLGAGLLWFGWFGFNVGSALSLNDVALTAFINTNIAAAASALTWMLSEWFFQSKPTAMGAACGVVSGLVAITPACGF
VTPFSALLIGAIGGVLCFGAVFFLKTKFGYDDTLDAFGCHGIGGTWGGIATGLFATTTVNADGANGLFYGNAALLFKQLVAIGATYAFTILMT
YAIIKAINYFLPVRVDEHEEHMGLDISMHGEKAYEYTERVN
>*Homo_sapiens*_Rh_type_B
MNFTFATQKSLTLLPRLECNGAISAHCNLHLPGFQDVHAMVFVGFGFLMVFLQRYGFSSVGFTFLLAAFALQWSTLVQGFLHSFHGGHIHVG
VESMINADFCAGAVLISFGAVLGKTGPTQLLLMALLEVVLFGINEFVLLHLLGVRDAGGSMTIHTFGAYFGLVLSRVLYRPQLEKSKHRQGSV
YHSDLFAMIGTIFLWIFWPSFNAALTALGAGQHRTALNTYYSLAASTLGTFALSALVGEDGRLDMVHIQNAALAGGVVVGTSSEMMLTPFGA
LAAGFLAGTVSTLGYKFFTPILESKFKVQDTCGVHNLHGMPGVLGALLGVLVAGLATHEAYGDGLESVFPLIAEGQRSATSQAMHQLFGLFV
TLMFASVGGGLGGLLLKLPFLDSPPDSQHYEDQVHWQVPGEHEDKAQRPLRVEEADTQA
>*Rattus_norvegicus*_Rh_type_C
MAWNTNLRGRLPITCLILQVTMVVLFGVFVRYDIQADAHWWLEKKRKNISSDVENEFYYRYPSFEDVHAMVFVGFGFLMTYLQRYGFSAVG
FNFLLAAFGIQWALLMQGWFHFFEEGHILLSVENLIQADFCVASTCVAFGAVLGKISPMQLLIMTFFQVTLFTVNEFILLNLIEAKDAGGSMTIH
TFGAYFGLTVTWILYRKNLEQSKQRQSSVYHSDLFAMIGTLFLWIYWPSFNSASSFHGDTQHRAALNTYLSLAASVLTTVAVSSVIHKKGKLD
MVHIQNATLAGGVGVGTAAEMMLTPYGALIVGFFCGILSTLGFAYLSPFLESRLRIQDTCGIHNLHGIPGIIGGIVGAVTAAYSSPDVYGEPGIV
HSFGFGGYKADWTKRMQGRSQIFGLLLSLAMALVGGIIVGFILKLPFWGQASDENCFEDAIYWEVPEEVNTVYIPEDLAHKHSTSLVPAIPLVL
STPSASIVPPVPPTPPASLATVTSSSLVH
>*Danio_rerio*_Rh_type_B
MAESTNLRLRLPLICIILEVILIILFGVLVEYNDDTDAKKWNKNNSTDPATNEFYYRYPSFQDVHVMIFVGFGFLMTFLQRYGFSSMGFNFLIAA
FSLQWATLMQGFFHGMHHGKIHVGVTSMINADFCTGAVLISFGAVLGKTSPVQLLVMAILEVTLFAVNEYILLSILGANDAGGSMTIHTFGAY
FGLMVTRILHRPNLDKSKHKNSSVYHSDLFAMIGTIFLWMFWPSFNSAITQYGDPQHRTAANTYYSLAACTLATFGFSSLVNPEGKLDMVHIQ
NAALAGGVAVGTAGEMMLTPFGSMIVGFLAGTISVLGYKYLTPFMESKLKIQDTCGIHNLHGMPGILGAIVGAVTAALASRDVYGNGLDKVF
LEAADNSQWSAQTKGGFQAISLAVTLGIALIGGLITGFLLKLPIYGTPPDTQCFEDAVYWEVPGEEEDHHELNEVSTQNEVEKLNS
>*Kryptolebias_marmoratus*_Rh_type_C
MGNCCQYMGPQKNTYVRVSLPAVCFVWQIAMVILFGVFIRYDKESDTHWVEHKKHENLTDLDNDFYFRYPSFQDVHVMIFVGFGFLMTFLK
RYSYGGVGFNFLIASFGLQWALLMQGWFHSPDPATGKIYIGVESLINADFCCAGSLIAFGALLGKVSPVQTMVVTLFGFTLFAVEEYIILDLLH
CRDAGGSMVIHCFGGYYGLAISWMLYRPNLHQSKNLHGSIYHSDLFAMIGTLFLWMFWPSFNSAITDHGDGQHRAAINTYIALASSVLTAVAI
SSLAHKKGKLDMVHIQNATLAGGVAMGTAAEFMITPYGALIVGFCMGIISTFGYLFITPFLEKYLKLQDTCGVHNLHAVPGMLGGFIGAIVAA
AASTEVYSEEGLINTFDFEGKYANRGVGTQGGYQAAGTCVSIAFGLVGGALVGLILKFPIWGDPADDNCFDDEAYWELPEEEEDILPPVLEYN
NHMINKQDIAESNFTMEET
>*Takifugu_rubripes*_Rh_type_A
MPAYATNMRLKFPILALTLELLTIVLFAVFVVYDDGKPSSDPHDPHDPHAGNHTQEGAPMDLYPMFQDVHVMIFIGFGFLMTFLKRYGFSSV
GVNLLLAAFGLQWGLLMQGFWHMEDGKIKINIFKIINADFSTATVLISFGAVLGKTSPVQLLIMTILEITIFSINEHLVATVIHANDVGASMIIHA
FGAYFGLAVARVLYRPGLKNGHEKEGSVYHSDMFAMIGTVFLWMFWPSFNSAIADPGFTQLTAVINTYLSLAACVLSAYAVSSLVEHKGKL
DMVHIQNATLAGGVAVGTCADMNIGPFGAMLIGLVAGIISTLGFKFLTPILASSLGIQDTCGVHNLHGMPGILGGVAGIVAVAMGKKDGGNA
SMQAAALASSLGFALVGGLVTGFIMKIPLWGQPPDQNCYDDSLYWEVPDEEEESGESFAHADHSKNKAEV
>Ralstonia_pickettii_12J_Rh_type
MNSIKTGGDALFILLGAIMVLAMHAGFAFLELGTVRKKNQVNALVKILVDFAVSTIAYFFIGYSVAYGVNFFTGADVLAQQNGYELVKFFFLL
TFAAAIPAIISGGIAERSRFEPQLAATFVLVGFVYPFFEGIAWNERFGIQHWLQSVTGAEFHDFAGSVVVHAVGGWIALPAVLLLGARHGRYKD
DGRIAAHPPSNIPFLAAGAWVLAVGWFGFNVMSAQTLDKMSGLVAINSLMAMVGGTLTAWWMGKNDPGFSYNGPLAGLVAVCAGSDVM
HPLGALAVGGIAGVLFVWMFTRVQNVWRIDDVLGVWPLHGLCGAWGGIAAGIFGLKALGGLGGVSFWAQVAGTAGGIVIALVGGTVVYGT
LRKLVGLRLDREAEFMGADLAIHRVSSTPESETHW
>Burkholderia_ambifaria_IOP40-10_Rh_type
MDGLKSGVDTLFLLIGAVMVLAMHAGFAFLELGTVRKKNQVNALVKILVDFSVSTLAYFFIGYTIAYGVEFFDDIGTLSQHNGYALVRFFFLL
TFAAAIPAIVSGGIAERAKFNPQLVATLIIVGFIYPFFEGIAWNDRFGVQDWLTHAFGAPFHDFAGSVVVHAFGGWVALPAVLLLGARHGRYA
KDGRIAAHPPSNIPFLALGAWVLAVGWFGFNVMSAQTLDKISGLVAVNSLMAMVGGTLAAWMAGRNDPGFTYNGPLAGLVAVCAGSDVM
HPIGALVTGAAAGAVFVAMFTCVQNKWRIDDVLGVWPLHGMCGALGGLAAGVFGQPVFGGLGGVSFVSQLIGTLGGIAIATAGGTLVYGAL
KATVGLRLDREAEFDGADLSIHRISATPERD
>Pseudomonas_putida_HB3267_Rh_type
MENMHSAMDSLVHGSNTLFILMGAILVLAMHAGFAFLEVGTVRHKNQVNALSKILSDFAISALVYFFIGYWIAYGVSFFQPAAALAADHGYA
LVKCFFLLTFAAAIPAIISGGIAERARFVPQLCATALIVAFIYPFFEGVVWNGNLGVQAWLQARFGAPFHDFAGSVVVHAMGGWLALAAVLLL
GARRGRYRDGRLVAFAPSSIPFLALGSWILIIGWFGFNVMSAQTLQGVSGLVAINSLMAMVGGTLSALLAGRNDPGFLHNGPLAGLVAVCAG

SDLMHPIGALATGLVAGALFVWTFTAAQNRWKIDDVLGVWPLHGLCGVWGGMACGIFGQEVLGGMGGVSLVSQLLGSLMGVVVALAGGF
AVYGAIRALHGLRLSHEQEFQGADLSLHRIGATSQD
